# Is remotely supervised ultrasound (tele-ultrasound) inferior to the traditional service model of ultrasound with an in-person imaging specialist? A systematic review

**DOI:** 10.1186/s13089-025-00440-6

**Published:** 2025-07-28

**Authors:** Thy Lai, Tania Stafinski, Jeremy Beach, Devidas Menon

**Affiliations:** 1 Health Technology & Policy Unit, School of Public Health, University of Alberta, Edmonton, AB Canada; 2 College of Physicians & Surgeons of Alberta, Edmonton, AB, Canada

## Abstract

**Background:**

Tele-ultrasound is known to offer potential benefits such as improved access and cost efficiency, but concerns still exist about image quality, operator skill, and data security. This study aimed to determine whether remotely supervised ultrasound is inferior to traditional in-centre ultrasound with an in-person imaging specialist regarding patient care quality, service quality, and access to care.

**Methods:**

A systematic search for a critical appraisal of relevant peer-reviewed published literature, as well as a jurisdictional scan of relevant regulations and standards in other Canadian jurisdictions, was performed.

**Results:**

Of the original 6051 discrete records identified through the search, 18 studies were selected for inclusion in the review. They originated from 11 countries, and the patient populations spanned infants, children, adults, and pregnant women. The medical applications were echocardiography (including fetal), obstetrical ultrasound, breast ultrasound, thyroid ultrasound, and abdominal ultrasound. The distance between the tele-ultrasound site and the reference site ranged from 23 to 365 km, or a 30 to 45-min drive. In 3 studies, tele-ultrasound images were acquired in one country (India, Peru) and interpreted in another (US or UK). The majority of studies reported good diagnostic accuracy (the proportion of agreement between tele-ultrasound and in-centre ultrasound ranged from 43.4% to 100%, sensitivity ranged from 43% to 97%, and specificity ranged from 77.4% to 100% across studies and tele-ultrasound application). Details are displayed in Supplementary Table 2. There was limited evidence on patients’ and providers’ perspectives on tele-ultrasound, but in the studies identified, more than half of the patients surveyed felt that tele-ultrasound was acceptable. Additionally, all comments from providers were positive, including their perspectives on the value of tele-ultrasound. The image quality results were mixed. Some studies found that image quality ranged from at least sufficient quality for diagnosis to excellent. However, some other studies reported inadequate image quality in up to 36.8% of cases. It is possible that this range of responses may be due to the varying technical ability/capacity of local tele-ultrasound systems to acquire and transmit images to a remote reader. Cost savings associated with tele-ultrasound were also reported and attributed mainly to travel costs for patients.

**Conclusion:**

There is no consistent evidence that tele-ultrasound is inferior to in-centre ultrasound, although further high-quality studies are needed.

**Supplementary Information:**

The online version contains supplementary material available at 10.1186/s13089-025-00440-6.

## Background

Tele-ultrasound has been defined as “the use of ultrasound with voice and video and an additional instructor, such as an ultrasound-certified physician, who is remotely connected to it” [1]. Tele-ultrasound was first used in the 1960s, when scans were performed on US astronauts with guidance from Mission Control. Since then, efforts have been made to further develop the technologies involved in tele-ultrasound, and numerous applications have been identified. A review of published studies in 2022 identified strengths and opportunities, including the practicality of performing tele-ultrasound (usability in both rural and urban areas), cost efficiency, and its application in medical education. Potential challenges included the ability of operators, image quality, and the safety of personal data [2]. The College of Physicians and Surgeons of Alberta, the provincial professional regulator, commissioned this project to inform the development of standards for tele-ultrasound. The College excluded point-of-care ultrasound (POCUS) from the project, as the standards being contemplated did not cover POCUS.

## Methods

The methods comprised (a) a systematic search for and critical appraisal of relevant peer-reviewed published literature and a qualitative synthesis of findings, and (b) a jurisdictional scan of relevant regulations and standards in other Canadian jurisdictions.

### Literature review

A systematic review of relevant scholarly work was conducted following internationally recognized published methodological guidelines. This comprised the following steps.

- *Identification of relevant papers*: A comprehensive, systematic search for relevant published literature was undertaken using structured search strategies applied to the following databases: PubMED, The Cochrane Library, Centre for Reviews and Dissemination (DARE, HTA and NHS EED), EMBASE, EMCARE, Web of Science, Scopus, Proquest, Econlit, JSTOR, and CINAHL. The structured search strategies were developed in collaboration with a health information specialist/research librarian and included relevant controlled vocabulary terms (Medical Subject Headings (MeSH)) and keywords. The search strategy is attached as Supplementary Material A—Search strategy. The searches were restricted to English-language literature. For completeness, a manual search of reference lists of included papers was also undertaken. All of the search results were entered into EndNote® reference management software, and duplicate citations were removed.
- *Selection of included studies*: Two researchers independently screened all titles and abstracts of citations using the inclusion and exclusion criteria in Table 1. As needed, they met to compare results, resolve any discrepancies, and select potentially relevant citations for retrieval. They then independently reviewed the corresponding full papers using the same criteria and met to compare results. Disagreements were resolved through discussion. No third-party review was necessary.

**Table 1:** Inclusion and exclusion criteria

Parameter	Inclusion criteria	Exclusion criteria
Population	Patients of any age group (e.g., neonates, children, adults) who require diagnostic ultrasound services	Populations who do not require ultrasound services as part of their care Volunteer patients Experimental or simulation-based tele-ultrasound without patient involvement
Intervention	Tele-ultrasound	Point-of-care ultrasound Ultrasound as a screening tool
Comparator	Traditional in-person ultrasound services	
Outcomes	Diagnostic accuracy Patient outcomes Patient satisfaction Provider satisfaction Service delivery times	Technical performance
Study design	Studies comparing tele-ultrasound to traditional in-person ultrasound Studies conducted in various healthcare settings, including hospitals, clinics, rural and remote areas, and low-resource settings	Studies focusing solely on the technical development of tele-ultrasound without addressing clinical or service outcomes Studies conducted in experimental, lab, or simulated settings

**Fig. 1:**
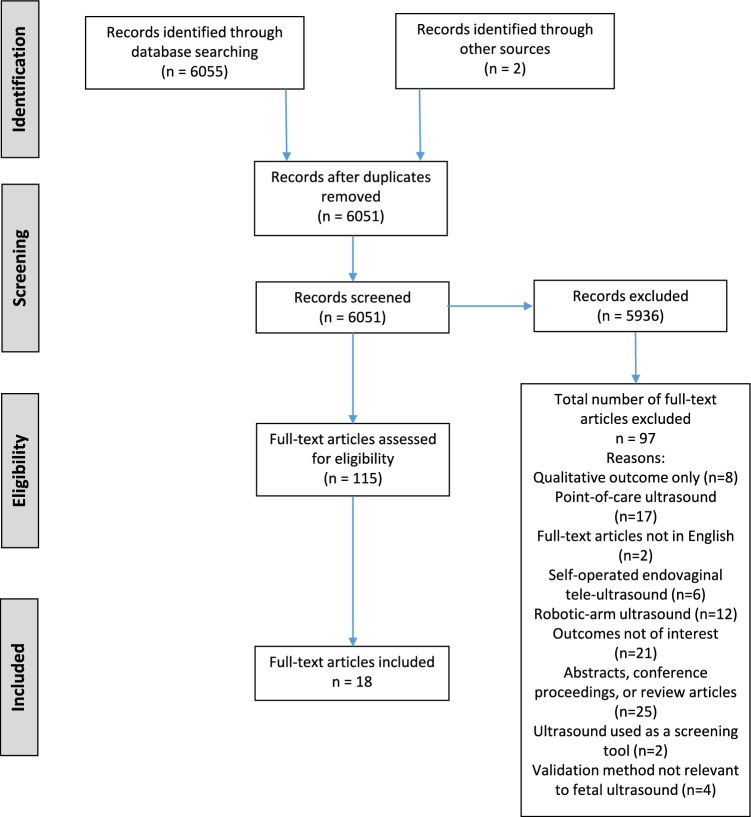
PRISMA flow diagram

The original searches yielded 6,051 discrete records, which were reduced to 18. The study selection process is illustrated in Fig. 1 (PRISMA flow diagram). This diagram also presents the reasons for the exclusion. They were: POCUS; reference test used to validate fetal tele-echocardiography was postnatal imaging; or tele-ultrasound was used for screening purposes rather than diagnosis.

- *Data extraction*: Systematic data extraction from the 18 included papers was done using a standardized data abstraction form/template. The form/template included the following information: author(s), publication year, country of origin, study type, study quality, purpose, design, setting, interventions, study population, intended outcome (e.g., detection of fetal abnormalities), outcomes measured, findings, and limitations. One researcher extracted data from each paper and a second researcher verified the contents of the form. For quality assurance, data from 10% of the papers were extracted by both researchers and compared to identify and, if necessary, resolve any discrepancies. For the remaining papers, one researcher extracted the data, and the other independently verified the extracted information to ensure accuracy and consistency.
- *Critical appraisal*: The methodological quality of randomized studies and non-randomized studies was independently assessed by two researchers using the QUADAS-C tool [3]. Endorsed by the Cochrane Group, the QUADAS-C is a generic tool for appraising the quality of studies of diagnostic test accuracy. It contains four domains, each of which evaluates a different risk of bias: (1) Patient selection—whether patients were selected in a way that could have introduced bias, (2) Index test (the test being evaluated)—whether it was interpreted without knowledge of the reference standard (blinding) and applied consistently across participants, (3) Reference standard (the gold standard test used for comparison)—whether it was correctly applied and interpreted independently of the index test, and (4) Flow and timing—whether all patients received the same reference standard, any patients were excluded from analysis in a way that could have introduced bias, and the time interval between index and reference tests was appropriate. Individual studies are scored on each domain in 3 categories: high, low, or unclear risk of bias. Discrepancies were resolved through discussion to reach consensus, without the involvement of a third reviewer. To assess inter-rater reliability, Cohen’s kappa (κ) coefficients were calculated for each domain prior to consensus. Kappa values were computed using Microsoft Excel based on observed and expected agreement, derived from cross-tabulations of the reviewers’ independent ratings (low, high, or unclear risk of bias) across all included studies. This measure quantifies agreement beyond chance, which provides an objective evaluation of consistency between reviewers.

### Jurisdictional scan

Each medical regulatory college in the 10 Canadian provinces and 3 territories was contacted via email to request an interview to collect information on existing practices, guidelines, or standards, and any geographic (i.e., distance) restrictions related to the delivery of tele-ultrasound. Contacts were identified with assistance from the staff of the College of Physicians and Surgeons of Alberta.

## Results

### Description of included studies

Studies were published between 1996 and 2022, and included 11 countries (China, Ethiopia, India, Japan, Norway, Peru, Spain, Switzerland, the United Kingdom, and the United States). Patients included infants, children, adults, and pregnant women, and the number ranged from 9 to 774. The study period ranged from 2 months to 5 years. The medical applications included echocardiography (including fetal) (12 studies), obstetrical ultrasound (2 studies), breast ultrasound (1 study), thyroid ultrasound (2 studies), and abdominal ultrasound (1 study). Across studies, there were considerable variations in the methods used and outcomes measured. A summary of the characteristics of the included studies is provided in Supplementary Material Table 1. Definitions of technical terms and abbreviations used in the tables are provided in Supplementary Material B—Glossary and Abbreviations.

For the purposes of this paper, the tele-ultrasound “arm” is labeled as “Index test 1”. The “Reference test” is the gold standard, and usually an on-site ultrasound by an expert. All comparisons described in this review are between the Index test 1 and the reference test, although some studies utilized a second index test (e.g., videotapes of images or ultrasounds performed and interpreted by trainees/physicians who were not experienced in ultrasound).

With regards to the distance between the tele-ultrasound site and the reference site, individual studies reported distances between 23 and 365 km, 75 km, 100 km, 200 km, 120 km, and in one case, a 30- to 45-min drive. In three studies, tele-ultrasound images were acquired in one country (India, or Peru) and interpreted in another (the US, or the UK).

Personnel involved in the acquisition of tele-ultrasound images included experienced sonographers, obstetricians, or healthcare providers with limited ultrasound expertise, such as pediatricians, medical trainees, nurses, and resident physicians. While sonographers and obstetricians had formal training, other providers received targeted education to perform ultrasound examinations. Interpreters were radiologists or specialists with expertise in the relevant field, such as cardiologists or obstetricians. Most studies reported the use of real-time expert guidance and image interpretation.

The most common outcomes reported were: (1) diagnostic accuracy metrics (proportion of agreement between tests, sensitivity, specificity, positive predictive value, negative predictive value, intra-class correlation coefficients), (2) change in diagnosis/treatment after tele-ultrasound was performed, and (3) image quality of tele-ultrasound. Some studies also reported qualitative outcomes, such as patient's and health provider's considerations. These outcomes are summarized in Tables 2 and 3, with detailed diagnostic accuracy data available in Supplementary Material Table 2.

**Table 2:** Patient and provider perspectives

Study	Patient considerations (%)	Provider considerations
Grant et al. (2010) [11]	NR	Performers’ satisfaction: Telemedicine is useful: 4.5 ± 0.82 (out of 5), they felt reassured by the facility: 4.2 ± 1.09
McCrossan et al. (2011) [13]	NR	Performers’ satisfaction rate at the start /end of the study: average of 2.7/3.8 (out of 5)
Sun et al. (2022) [14]	Acceptance: yes—61.9%, uncertain—5.2%, no—33% Willing to pay: yes—60.6%, uncertain—11.1%, no—28.3%	Providers’ satisfaction: Value of tele-ultrasound in diagnosis—yes: 69.7%, uncertain: 1%, no- 29.3% Guidance had a training effect on the performer—yes—68%, uncertain—2%, no—29.3%
Li et al. (2022) [15]	Acceptance: yes—63.6%, uncertain—2%, no—34.3% Willing to pay: yes—59.8%, uncertain—4.1%, no—36.1%	Providers’ satisfaction: Guidance is helpful—yes: 62.9%, uncertain—2.1%, no—35.1% Guidance had a training effect on the performer: yes: 64.9%, uncertain—1%, no—34.0%
Jemal et al. (2022) [18]	Patients’ satisfaction: 96% felt comfortable during the procedure, 99% agreed that they would recommend to others, 98% would undergo another tele-ultrasound, 72% were satisfied with the image quality, 77% were satisfied with the sound quality, 36% were not comfortable communicating with remote obstetrician, 98% agreed that the encounter was private and confidential, 49% disagreed that they had to wait long to receive healthcare, 30% were unsure or agreed that they had to wait long, 76% agreed that they were given enough information to prepare for the ultrasound, and 63% agreed that they had enough time to think about questions and to ask the remote obstetrician	100% of providers felt: they had received adequate training for image acquisition, confident in their ability to obtain images, enjoyed using the system, felt their patients were satisfied with the care provided, improves access to services
Toscano et al. (2021) [19]	NR	Providers’ satisfaction: The confidence level of readers was 3 (out of 3) for all diagnoses
Evangelista et al. (2016) [20]	NR	Time saved by cardiologist: 4.2 h/week

**Table 3:** Image/audio quality

Studies	Quality measures
Lewin et al. (2006) [6]	Image quality: 94% excellent, 5% adequate, 0.4% unsatisfactory
Mulholland et al. (1999) [7]	Image quality: 97% of diagnostic quality
McCrossan et al. (2011) [13]	Median video quality 4/5, median audio quality 4/5, median overall quality 4/5
Sun et al. (2022) [14]	Image quality: 25.2% perfect, 50.1% minor improvement possible, 3% poor quality, 1% undiagnosable
Li et al. (2022) [15]	Image quality: 23.7% excellent, 46.4% good, 22.7% flawed but usable for diagnosis, 4% not good enough, 1% poor and cannot be used for diagnosis
Marini et al. (2021) [16]	Image quality: 87.6% excellent, 12.4% acceptable
Marini et al. (2021) [17]	Image quality: 24.3% excellent, 38.9% acceptable, 36.8% poor
Toscano et al. (2021) [19]	Image quality: 61.1% excellent, 38.1% acceptable, 0.8% poor
Evangelista et al. (2016) [20]	Image quality: 34% good, 45.4% acceptable, 19.2% poor, 8.7% inconclusive

Ten studies reported kappa (κ) scores to evaluate agreement between tele-ultrasound and in-person ultrasound by an expert, using Cohen’s kappa, κ [4]_._ However, in all but one, the two interpreters did not examine the same ultrasound image (instead, they compared interpretations of images obtained by different means). Although modified scores were proposed by other authors, e.g., Nelson and Edwards [5], which would have been more appropriate, there was no evidence that in the nine studies, they were used. In the remaining study, the value of κ was calculated between the experts who interpreted the same tele-ultrasound images. Intra-class correlation coefficients (ICCs) were also used to evaluate the agreement on continuous variables (ultrasound indices). Only one of these studies reported using a two-way random-effects model for calculating ICCs.

### Critical appraisal of studies

**Fig. 2:**
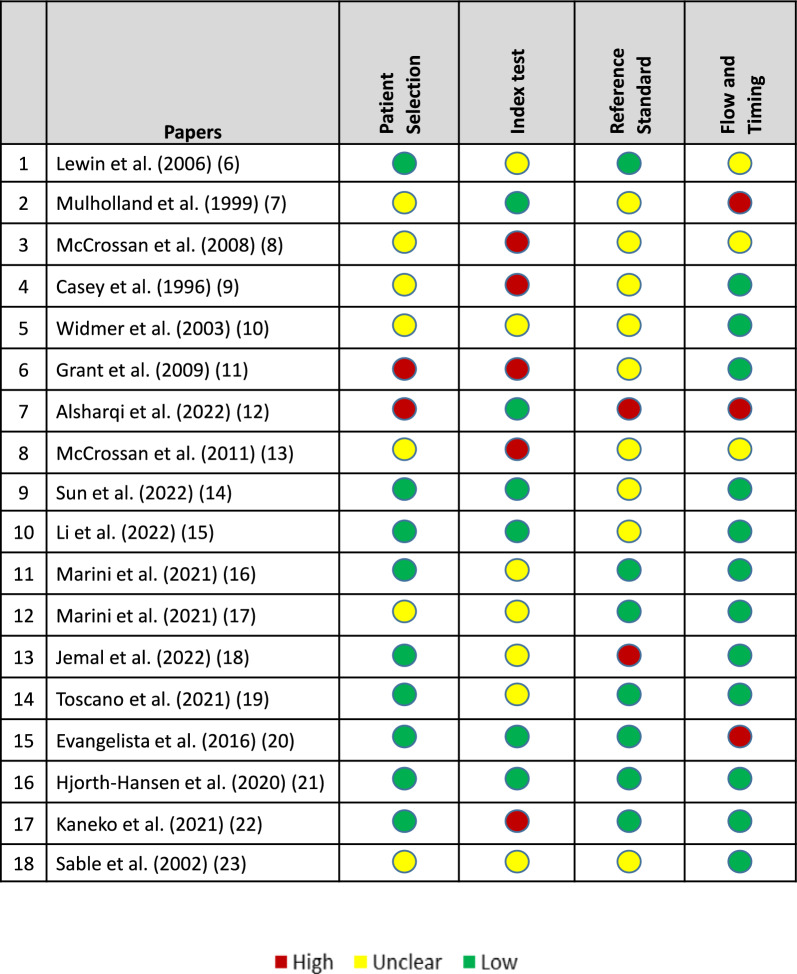
Summary of the quality of evidence of individual studies

**Fig. 3:**
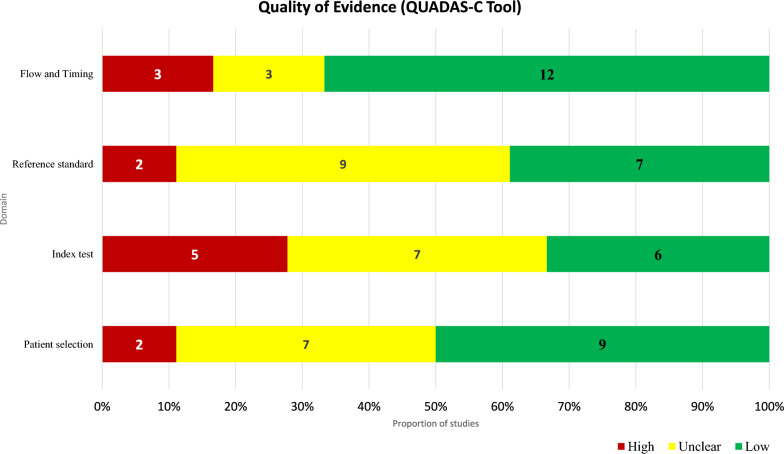
Variation in proportion of studies with different risks of bias across domains

The results of the critical appraisal of studies are described in Figs. 2 and 3. "Flow and timing" had the highest proportion of low-risk studies, with 12 studies classified as low risk and only two as unclear. Conversely, "Reference standard" had the greatest number of unclear-risk studies (9 studies). Both "Patient selection" and "Index test" had a more balanced distribution between unclear and low-risk classifications; however, they also showed a notable number of studies with a high risk of bias. The "Index test" exhibited the greatest number of studies with a high risk of bias (5 studies). Overall, only one study was rated as low risk of bias across all four domains, indicating that the majority of included studies had at least one methodological limitation.

The kappa values indicated high inter-rater agreement across all domains. Specifically, κ = 0.736 for Patient Selection (substantial agreement), κ = 0.831 for Index Test (almost perfect agreement), and κ = 1.000 for both Reference Standard and Flow and Timing (almost perfect agreement) [4]. These results reflect high inter-rater reliability and support the robustness of the risk-of-bias assessments.

### Results by outcome

Supplementary Material Table 2 and Tables 2 and 3 summarize the results of included studies by outcome.

Supplementary Material Table 2 contains the information on diagnostic accuracy measures reported in each study.

### Diagnostic accuracy

The *proportion of agreement* between tele-ultrasound and standard ultrasound refers to the proportion of cases in which both modalities yielded the same diagnostic results. In 11 of the 14 studies that reported this measure, agreement ranged from 86 to 100% [6–16]. Of the remaining three studies, the first reported values ranging from 43.4% to 94%, and agreement was considerably lower when visualising the gallbladder (70.1%), diagnosing an abnormal right kidney (65.2%), or diagnosing an abnormal pancreas (43.4%) [17]. The second study reported agreement rates ranging from 79 to 100% [18]. Except for the 79% agreement in placental grading, all other 14 diagnoses had agreement rates of 94% or higher. The third study reported values ranging from 76.2% to 100%, with 76.2% for confirmation of a live fetus based on cardiac signs [19]. In this study, the agreement was 85.6% or higher for the diagnoses reported.

*Sensitivity* was reported (or calculated from the data presented in the study) in 8 studies, with 6 demonstrating a sensitivity of 84% or higher [7, 8, 11, 13, 15, 17]. The other 2 studies reported lower values for sensitivity. One study reported a sensitivity of 62.5% for detecting left atrial dilation and 76% for identifying dilation of the aortic root of the proximal ascending aorta [20]. In another study, the sensitivity for detecting at least moderate aortic stenosis was reported as 43% (based on 7 analyzed cases) [21].

*Specificity* data were also available from 8 studies. The specificity was 92% to 100% [7, 8, 11, 13, 17, 20, 21], except in one study, where it was 77.4% [15].

*Positive predictive values (PPV)* were reported (or calculated from the data in the study) in 6 studies, with five of them demonstrating PPV values of 89% or higher [7, 8, 11, 13, 15]. In one study [20], the PPV was below 80% for 8 out of 10 diagnoses. However, for mitral regurgitation and hypertrophic cardiomyopathy, the PPV was 83.5% and 84.9%, respectively.

Six studies reported negative predictive values (NPV) ranging from 82 to 100% [7, 8, 11, 13, 20], while one study reported a lower NPV of 77.4% [15].

*Inter-observer agreement* [Intraclass correlation coefficients (ICC)] in tele-ultrasound varied depending on the clinical application. For tele-echocardiography, most echocardiographic indices demonstrated good agreement, although variation was observed in specific measurements. Mitral early diastolic velocity showed excellent agreement (ICC = 0.94), while tricuspid annular plane systolic excursion (TAPSE) had a considerably lower agreement (ICC = 0.44) [22]. Fetal biometry assessments in obstetrical ultrasound also exhibited differences in agreement depending on gestational age [19]. In the second trimester, most measurements exhibited good agreement, but abdominal circumference only demonstrated moderate reliability (ICC = 0.67) [19]. In the third trimester, overall agreement declined, with ICC values ranging from 0.28 to 0.38 for most parameters. However, estimated gestational age (ICC = 0.64) and femur length (ICC = 0.68) maintained moderate agreement. Regarding all gestational periods, agreement was good to excellent, with that for abdominal circumference (ICC = 0.81) at the lower end and that for gestational age (ICC = 0.95) at the upper end. With respect to thyroid ultrasound, agreement varied across different aspects of assessment. Measurements of thyroid lobe diameters showed poor agreement, except for those relating to transverse diameters, which demonstrated moderate agreement (ICC = 0.57–0.58) [16]. In another study on thyroid ultrasound, classification using the TI-RADS categories and evaluation of nodule features exhibited good agreement, while nodule measurements showed excellent agreement [15]. Similar patterns were observed in breast ultrasound, where BI-RADS categories and target nodule measurement achieved excellent agreement. However, certain parameters related to nodule features only showed moderate agreement [14].

*Changes in medical management* were examined by comparing tele-ultrasound interpretations to initial decisions made by physicians performing ultrasound without expert consultation. Changes in diagnosis resulting from the comparison of tele-ultrasound and in-person ultrasound by an expert (Reference test) address the diagnostic accuracy of tele-ultrasound; therefore, they were not reported (see paragraph on *Proportion of agreement* above). Three studies on tele-echocardiography demonstrated the impact of expert interpretation on treatment modifications and the necessity of urgent transfers to tertiary hospitals. Consultation with specialists significantly reduced unnecessary patient transfers, with one study indicating that 72% of transfers were avoided due to expert interpretation [8]. In another study, 5% of cases were urgently transferred and 30.2% initial treatment plans were altered following expert review of tele-ultrasound images [23]. The third study elaborated on the adjustments in family doctors’ initial management strategies after consultation with cardiologists: 75% of patients did not require conventional echocardiography, 61% did not need referral to cardiology, 42% did not need clinical follow-up, and 48% should not have been discharged [20].

### Patient and provider perspectives

Table 2 presents a summary of information on the perspectives of patients and ultrasound providers on tele-ultrasound. Seven of the 14 studies provided varying amounts of data on these aspects of tele-ultrasound.

*Patient considerations* Three studies sought patients’ opinions using surveys. Overall, patients were relatively positive about tele-ultrasound. In two of these studies, approximately 60% or more of patients found tele-ultrasound to be acceptable and the same amount were willing to pay for the test [14, 15]. However, about a third of them did not find the test to be acceptable. It is important to note that these studies were conducted in China, where differences in the healthcare financing mechanism may have influenced the findings. In the third study, while privacy and confidentiality were widely acknowledged, some patients reported challenges with communication, image and sound quality, and perceived wait times [18].

*Provider considerations* Seven studies reported on some aspects of providers’ opinions (on utility and satisfaction) regarding tele-ultrasound. All the comments were positive, including confidence in using the system and the value of tele-ultrasound.

### Image and audio quality

Table 3 provides a summary of findings on image quality, which were reported in 9 of the 18 studies reviewed. Almost all of these studies concluded that images were “excellent” or at least of diagnosable quality. However, images in some studies rated as “poor”, “inconclusive”, “unsatisfactory” or “undiagnosable” ranged from 0.4% to 36.8%. These variations were likely due to the differences in how each category was defined across various studies and the technical capacity of the various systems used to acquire and transmit images.

### Other findings

*Time to perform tele-ultrasound* varied depending on the type of ultrasound. Regarding echocardiography, two studies reported that the mean performance time, including consultation time with remote experts, was approximately 70 min (range 60–79.2 min) [21, 23]. Abdominal volume sweep imaging was reported to take approximately 10 min in another study of abdominal imaging [17]. Two other studies reported the mean performance time for breast and thyroid ultrasound to be 6.6 and 4.6 min, respectively [14, 15].

*Cost savings* were reported in 3 studies. The first study, from 1999, estimated cost savings based on a 74% reduction in patient transfers, preventing 47 ambulance trips at approximately £300 ($480) each, resulting in a total savings of £14,100 ($22,560) over two years [7]. The second study, which was published in 2009, compared tele-ultrasound with standard care, demonstrating per-patient cost reductions through decreased ambulance transfers and in-person specialist consultations [11]. The total savings per patient were £1822, £608, and £739 across the three regional hospitals assessed. The third study, from 2022, reported a cost savings of 9.2 Ethiopian Birr (ETB) in travel expenses for patients accessing telemedicine sites compared to traveling to central hospitals [18]. Given that Ethiopia’s Gross National Income (GNI) per capita (Atlas method) in Ethiopia was $1010 US (130,492 ETB) in 2022 [24], the reduction in travel costs was relatively insignificant.

*Impact of distance on performance of tele-ultrasound* was addressed in 13 studies. However, they did not consistently report on temporal remoteness, providing information on distances (or traveling times) only between the tele-ultrasound and in-centre sites only. In six of these studies, the distance reported ranged from 30 to 346 km. In three, the tele-ultrasound and in-centre sites were named the same as in the first six studies, so the distance range would have been the same. In the remaining three studies, the tele-ultrasound site was in one country (India/Peru) and the expert was in another (US/UK). Again, the diagnostic accuracy was good or excellent (proportion of agreement in the 90% range, sensitivity and specificity in the 84% to 100% range). Overall, there was no good evidence of distance being a factor in diagnosis. There was one possible exception [20], where the study reported sensitivity for 2 measures (aortic root or proximal ascending aorta, and left atrium dilation) of 76% and 62.5%, respectively, while the values for the 8 other measures were 80% to 100%.

### Jurisdictional scan

The jurisdictional scan of other jurisdictional colleges of physicians and surgeons yielded relatively scant information. Two jurisdictions (Manitoba and Quebec) had relevant information and consented to an interview. Table 4 contains a summary of information from Manitoba and Quebec. In both cases, the distance between a tele-ultrasound site and a central imaging centre was not identified as a consideration in the permitted use of tele-ultrasound, and no geographical distance restrictions on tele-ultrasound were reported. In the other jurisdictions, the response was that there were no jurisdictional regulatory standards in place relating directly to tele-ultrasound.

**Table 4:** Jurisdictional scan

Jurisdiction	Geographical restrictions	Training requirements
Manitoba	No, as long as the remote sites have accredited ultrasound machines and trained operators	The person who acquires tele-ultrasound images must be a certified sonographer or have equivalent qualifications
Quebec	No	There are additional training and requirements for non-physician professionals (notably technologists) who perform ultrasound examinations without the immediate review of the radiologist

## Discussion

According to our findings, tele-ultrasound can achieve diagnostic accuracy comparable to conventional ultrasound across several clinical applications. Most studies reported acceptable to high image quality, with minimal impact from the geographic distance between sites. Although some concerns regarding audio-visual quality and training needs were noted, patient and provider satisfaction were generally high. These results suggest the feasibility of tele-ultrasound as an alternative to conventional ultrasound to improve access to diagnostic imaging in underserved settings, particularly with the support of remote experts. While real-time (synchronous) tele-ultrasound might often be considered advantageous due to the possibility of immediate expert feedback and probe adjustment, our review could not determine whether it consistently leads to higher diagnostic accuracy compared to asynchronous methods. This is primarily due to heterogeneity in study design, clinical settings, operator experience, and outcome reporting, which excluded direct comparisons. Further research is recommended to clarify the benefits of each approach.

Furthermore, the methodological quality of the included studies varied. Only one study demonstrated low risk of bias across all domains, while the majority had at least one domain rated as unclear or high risk. These limitations reduce the overall certainty of the evidence and suggest that, while the results are promising, they should be interpreted with caution.

Our review is different in scope and methodology from existing literature evaluating tele-ultrasound in Canadian and global contexts. Britton et al. [25] conducted a systematic review on tele-ultrasound in 2019 and also analyzed the clinical impact of tele-ultrasound. Their review was limited to low-resource areas in low-middle income countries (LMICs) (e.g., Togo, Uganda, Serbia) while our study included ones conducted in LMICs and also remote areas of resource-abundant countries (e.g., the UK). Additionally, they included feasibility studies, while in contrast, our review only focused on studies that assessed diagnostic accuracy, patient and provider satisfaction, and patient outcomes. While both reviews aimed to evaluate clinical utility, only our review synthesized diagnostic performance using standardized accuracy metrics (e.g., sensitivity, specificity, and ICCs), which results in a more robust evaluation of tele-ultrasound's equivalence to conventional imaging.

In addition to the systematic review, two recent Canadian studies explored tele-ultrasound in specific contexts [26, 27]. Both studies reported patient and provider satisfaction, as well as highlighted the potential of tele-ultrasound to improve access to care in underprivileged areas. Despite some shared findings, they differed from the studies included in our review in terms of design, focus, or population. One study in British Columbia evaluated the feasibility of a novel mixed-reality tele-ultrasound system in research environments with healthy volunteers, which limited clinical generalizability [26]. The study focused on human–computer interaction, latency, and system usability, rather than diagnostic performance or clinical outcomes. Notably, both this study and our systematic review arrived at the conclusion that long geographic distance did not adversely affect image quality. Another study in Alberta implemented a maternal–fetal medicine tele-ultrasound program with an emphasis on training, triage, and patient experience [27]. However, they lacked a comparator group (conventional ultrasound) for a formal assessment of diagnostic accuracy. Nonetheless, they pointed out similar benefits, such as improved access to ultrasound services and high patient and provider satisfaction, which aligned with our review’s findings. Although these studies did not meet our inclusion criteria, both studies offer informative insights into the implementation of tele-ultrasound in Canadian healthcare settings.

Our study poses some limitations. Some studies were not deemed eligible due to the inclusion of solely English literature. While many included studies demonstrated comparable diagnostic performance between tele-ultrasound and conventional imaging, the heterogeneity of clinical applications and outcome measures precluded a formal meta-analysis. Although meta-analyses improve precision, and offer an opportunity to address questions not addressed by the individual studies, they can lead to misleading results seriously. This can happen when the individual studies are very heterogeneous [28].

Among the studies reviewed for this project, there were variations in study designs, in the indications for the ultrasound, the time between the index test and the reference, and in outcomes that were inconsistently reported. Thus, findings were synthesized narratively. Many studies have small sample sizes and non-randomized designs, which might reduce the overall strength of the evidence. Although including studies from different countries and settings enhances the generalizability of our review, it also generates variability in equipment, provider training, healthcare systems, and implementation strategies. Additionally, regarding the jurisdictional scan, only 2 out of 13 medical regulatory colleges (Manitoba and Quebec) consented to provide information.

## Conclusions

The purpose of this review was to examine existing evidence to determine whether remotely supervised ultrasound (tele-ultrasound) has been shown to be inferior to the traditional service model of ultrasound with an in-person imaging specialist insofar as patient care quality, service quality, and access to care are concerned. In addition, it was to determine whether the geographical distance between the tele-ultrasound location and the in-centre ultrasound site impacted diagnosis.

This review concludes that there is no consistent evidence that overall, tele-ultrasound, with real-time guidance, is inferior to conventional in-centre ultrasound. However, in some cases (e.g., for breast or thyroid applications, there were too few studies to make a conclusive statement about tele-ultrasound. The evidence also demonstrates that the distance between the tele-ultrasound site and the expert in-centre interpreter of the images has no significant impact on the effectiveness of tele-ultrasound in diagnosis.

## Supplementary Information

Supplementary material 1.

Supplementary material 2.

Supplementary material 3.

Supplementary material 4.

Supplementary material 5.

## Data Availability

All data generated or analyzed during this study are included in this published article and its supplementary information files.
