# Supplementary material for: Is remotely supervised ultrasound (tele-ultrasound) inferior to the traditional service model of ultrasound with an in-person imaging specialist? A systematic review
Source: Ultrasound J. 2025 Jul 28;17:34. doi: 10.1186/s13089-025-00440-6 (PMC12304338; doi:10.1186/s13089-025-00440-6)
Supplement: Supplementary file 1 — Supplementary material 1. [file 13089_2025_440_MOESM1_ESM.docx]

**ABBREVIATIONS AND GLOSSARY**

***Abbreviations***

| **Abbreviation** | **Full Term** |
| --- | --- |
| AA | Aortic root or proximal ascending aorta |
| AP | Anterior - posterior |
| A-P | Anterior-posterior |
| AR | Aortic regurgitation |
| AS | Aortic stenosis |
| HCM | Hypertrophic cardiomyopathy |
| HCU | Hand-held cardiac ultrasound |
| HfmrEF | Heart failure with mid-range ejection fraction |
| HfpEF | Heart failure with preserved ejection fraction |
| HfrEF | Heart failure with reduced ejection fraction |
| LA | Left atrial |
| LAD | Left atrial diameter |
| LV | Left ventricle |
| LVD dysf | Left ventricular dysfunction |
| LVDIs | Left ventricular end-systolic diameter |
| LVH | Left ventricle hypertrophy |
| LVIDd | Left ventricular end-diastolic diameter |
| MR | Mitral regurgitation |
| MS | Mitral stenosis |
| NA | Not applicable |
| NR | Not reported |
| Per | Pericardial |
| RA | Right atrial |
| RHD | Rheumatic heart disease |
| RV | Right ventricle |
| TAPSE | Tricuspid annular plane systolic excursion |
| VSI | Volume sweep imaging |

***Glossary***

| **Abbreviation** | **Full Term** |
| --- | --- |
| Breast nodule ultrasound features and measurements | Characteristics assessed during imaging: shape, orientation, margin, echo pattern, posterior features, calcifications, vascularity, internal characteristics, and nodule dimensions (transverse, A-P, longitudinal) |
| Cardiovascular diagnoses | Includes: aortic valve stenosis/regurgitation, mitral valve stenosis/regurgitation, tricuspid valve stenosis/regurgitation, rheumatic valve disease, LV enlargement, LV dysfunction, LVH, RA/LA enlargement, pericardial effusion, thrombus, tachycardia |
| Echocardiographic indices | Quantitative ultrasound measures of cardiac structure/function: LVIDd, LVIDs, LAD, TAPSE, LVEF, mitral E/A ratio, E/e′ ratio, IVS/PW thickness, LA/LV volume and dimensions |
| Fetal biometry | Standard fetal measurements: biparietal diameter, head circumference, abdominal circumference, femur length, estimated gestational age |
| Thyroid lobe diameters | Linear measurements of each thyroid lobe: right/left lobe A-P and transverse diameter, and isthmus A-P diameter |
| Thyroid nodule features | Characteristics used to evaluate thyroid nodules: composition, echogenicity, shape, margin, echogenic foci, vascularity, and dimensions (transverse, A-P, longitudinal) |
