# Supplementary material for: Is remotely supervised ultrasound (tele-ultrasound) inferior to the traditional service model of ultrasound with an in-person imaging specialist? A systematic review
Source: Ultrasound J. 2025 Jul 28;17:34. doi: 10.1186/s13089-025-00440-6 (PMC12304338; doi:10.1186/s13089-025-00440-6)
Supplement: Supplementary file 2 — Supplementary material 2. [file 13089_2025_440_MOESM2_ESM.docx]

**The search strategy**

Ovid Multifile

Database: Ovid MEDLINE(R) ALL <1946 to May 14, 2024>, Embase <1974 to 2024 May 14>

Search Strategy:

--------------------------------------------------------------------------------

1 Telemedicine/ and exp Ultrasonography/ (1232)

2 (teleultrason* or tele-ultrason* or tele-ultra-son* or teleultra-son* or teleultrasound* or tele-ultrasound* or tele-ultra-sound* or teleultra-sound* or "tele US" or "tele U/S").tw,kw,kf. (278)

3 (TUS adj10 tele*).tw,kw,kf. (14)

4 (teleechogra* or tele-echogra* or teleechocardiogra* or tele-echocardiogra* or tele-echo-cardiogra* or teleecho-cardiogra* or teleechotomogra* or tele-echotomogra* or tele-echo-tomogra* or teleecho-tomogra*).tw,kw,kf. (176)

5 (teleendosonogra* or tele-endosonogra* or tele-endo-sonogra* or teleendo-sonogra*).tw,kw,kf. (0)

6 (tele* ultrason* or tele* ultra-son* or tele* ultrasound* or tele* ultra-sound* or "tele* US" or "tele* U/S").tw,kw,kf. (329)

7 (telesonogra* or tele-sonogra*).tw,kw,kf. (127)

8 tele* sonogra*.tw,kw,kf. (20)

9 ((remote* or virtual*) adj3 (ultrason* or ultra-son* or ultrasound* or ultra-sound*)).tw,kw,kf. (2914)

10 ((remote* or virtual*) adj3 (echogra* or echocardiogra* or echo-cardiogra* or echo-cardio-gra* or echocardio-gra* or echotomogra* or echo-tomogra* or echo-tomo-gra* or echotomo-gra*)).tw,kw,kf. (267)

11 ((remote* or virtual*) adj3 sonogra*).tw,kw,kf. (436)

12 (RTMUS adj10 (ultraso* or ultra-so*)).tw,kw,kf. (15)

13 (POCUS adj10 (remote* or tele* or virtual*)).tw,kw,kf. (154)

14 or/1-13 [TELE-ULTRASOUND] (5091)

15 exp Animals/ not Humans/ (16986804)

16 14 not 15 [ANIMAL-ONLY REMOVED] (3942)

17 16 use medall [**MEDLINE RECORDS**] (1779)

18 exp telemedicine/ and exp echography/ (2178)

19 (teleultrason* or tele-ultrason* or tele-ultra-son* or teleultra-son* or teleultrasound* or tele-ultrasound* or tele-ultra-sound* or teleultra-sound* or "tele US" or "tele U/S").tw,kw,kf. (278)

20 (TUS adj10 tele*).tw,kw,kf. (14)

21 (teleechogra* or tele-echogra* or teleechocardiogra* or tele-echocardiogra* or tele-echo-cardiogra* or teleecho-cardiogra* or teleechotomogra* or tele-echotomogra* or tele-echo-tomogra* or teleecho-tomogra*).tw,kw,kf. (176)

22 (teleendosonogra* or tele-endosonogra* or tele-endo-sonogra* or teleendo-sonogra*).tw,kw,kf. (0)

23 (tele* ultrason* or tele* ultra-son* or tele* ultrasound* or tele* ultra-sound* or "tele* US" or "tele* U/S").tw,kw,kf. (329)

24 (telesonogra* or tele-sonogra*).tw,kw,kf. (127)

25 tele* sonogra*.tw,kw,kf. (20)

26 ((remote* or virtual*) adj3 (ultrason* or ultra-son* or ultrasound* or ultra-sound*)).tw,kw,kf. (2914)

27 ((remote* or virtual*) adj3 (echogra* or echocardiogra* or echo-cardiogra* or echo-cardio-gra* or echocardio-gra* or echotomogra* or echo-tomogra* or echo-tomo-gra* or echotomo-gra*)).tw,kw,kf. (267)

28 ((remote* or virtual*) adj3 sonogra*).tw,kw,kf. (436)

29 (RTMUS adj10 (ultraso* or ultra-so*)).tw,kw,kf. (15)

30 (POCUS adj10 (remote* or tele* or virtual*)).tw,kw,kf. (154)

31 or/18-30 [TELE-ULTRASOUND] (5854)

32 (exp animal/ or exp animal model/ or exp animal experiment/ or nonhuman/ or exp vertebrate/) not (exp human/ or exp human experiment/) (12660808)

33 31 not 32 [ANIMAL-ONLY REMOVED] (5744)

34 33 use oemezd [**EMBASE RECORDS**] (3846)

35 17 or 34 [**BOTH DATABASES**] (5625)

36 remove duplicates from 35 (4028) [**TOTAL UNIQUE RECORDS**]

37 36 use medall [MEDLINE UNIQUE RECORDS] (1772)

38 36 use oemezd [EMBASE UNIQUE RECORDS] (2256)

***************************

Web of Science

| # | Search Query | Results |
| --- | --- | --- |
| 1 | teleultrason* or (tele NEAR/0 ultrason*) or (tele NEAR/0 ultra NEAR/0 son*) or (teleultra NEAR/0 son*) or teleultrasound* or (tele NEAR/0 ultrasound*) or (tele NEAR/0 ultra NEAR/0 sound*) or (teleultra NEAR/0 sound*) or "tele US" or "tele U/S" (Topic) | 176 |
| 2 | TUS NEAR/10 tele* (Topic) | 23 |
| 3 | teleechogra* or (tele NEAR/0 echogra*) or teleechocardiogra* or (tele NEAR/0 echocardiogra*) or (tele NEAR/0 echo NEAR/0 cardiogra*) or (teleecho NEAR/0 cardiogra*) or teleechotomogra* or (tele NEAR/0 echotomogra*) or (tele NEAR/0 echo NEAR/0 tomogra*) or (teleecho NEAR/0 tomogra*) (Topic) | 171 |
| 4 | teleendosonogra* or (tele NEAR/0 endosonogra*) or (tele NEAR/0 endo NEAR/0 sonogra*) or (teleendo NEAR/0 sonogra*) (Topic) | 0 |
| 5 | (tele* NEAR/0 ultrason*) or (tele* NEAR/0 ultra NEAR/0 son*) or (tele* NEAR/0 ultrasound*) or (tele* NEAR/0 ultra NEAR/0 sound*) or (tele* NEAR/0 "US") or (tele* NEAR/0 "U/S") (Topic) | 1150 |
| 6 | telesonogra* or (tele NEAR/0 sonogra*) (Topic) | 72 |
| 7 | tele* NEAR/0 sonogra* (Topic) | 14 |
| 8 | (remote* or virtual*) NEAR/3 (ultrason* or (ultra NEAR/0 son*) or ultrasound* or (ultra NEAR/0 sound*)) (Topic) | 1834 |
| 9 | (remote* or virtual*) NEAR/3 (echogra* or echocardiogra* or (echo NEAR/0 cardiogra*) or (echo NEAR/0 cardio NEAR/0 gra*) or (echocardio NEAR/0 gra*) or echotomogra* or (echo NEAR/0 tomogra*) or (echo NEAR/0 tomo NEAR/0 gra*) or (echotomo NEAR/0 gra*)) (Topic) | 132 |
| 10 | (remote* or virtual*) NEAR/3 sonogra* (Topic) | 243 |
| 11 | RTMUS NEAR/10 (ultraso* or (ultra NEAR/0 son*) or (ultra NEAR/0 sound*)) (Topic) | 5 |
| 12 | POCUS NEAR/10 (remote* or tele* or virtual*) (Topic) | 48 |
| 13 | #12 OR #11 OR #10 OR #9 OR #8 OR #7 OR #6 OR #5 OR #4 OR #3 OR #2 OR #1 | 3488 |

Public Affairs Index

| # | Query | Limiters/Expanders | Last Run Via | Results |
| --- | --- | --- | --- | --- |
| S13 | S1 OR S2 OR S3 OR S4 OR S5 OR S6 OR S7 OR S8 OR S9 OR S10 OR S11 OR S12 | Search modes - Find all my search terms | Interface - EBSCOhost Research Databases  Search Screen - Advanced Search  Database - Public Affairs Index | 5 |
| S12 | TI ( POCUS N10 (remote* or tele* or virtual*) ) OR AB ( POCUS N10 (remote* or tele* or virtual*) ) | Search modes - Find all my search terms | Interface - EBSCOhost Research Databases  Search Screen - Advanced Search  Database - Public Affairs Index | 0 |
| S11 | TI ( RTMUS N10 (ultraso* or (ultra W0 son*) or (ultra W0 sound*)) ) OR AB ( RTMUS N10 (ultraso* or (ultra W0 son*) or (ultra W0 sound*)) ) | Search modes - Find all my search terms | Interface - EBSCOhost Research Databases  Search Screen - Advanced Search  Database - Public Affairs Index | 0 |
| S10 | TI ( (remote* or virtual*) N3 sonogra* ) OR AB ( (remote* or virtual*) N3 sonogra* ) | Search modes - Find all my search terms | Interface - EBSCOhost Research Databases  Search Screen - Advanced Search  Database - Public Affairs Index | 1 |
| S9 | TI ( (remote* or virtual*) N3 (echogra* or echocardiogra* or (echo W0 cardiogra*) or (echo W0 cardio W0 gra*) or (echocardio W0 gra*) or echotomogra* or (echo W0 tomogra*) or (echo W0 tomo W0 gra*) or (echotomo W0 gra*)) ) OR AB ( (remote* or virtual*) N3 (echogra* or echocardiogra* or (echo W0 cardiogra*) or (echo W0 cardio W0 gra*) or (echocardio W0 gra*) or echotomogra* or (echo W0 tomogra*) or (echo W0 tomo W0 gra*) or (echotomo W0 gra*)) ) | Search modes - Find all my search terms | Interface - EBSCOhost Research Databases  Search Screen - Advanced Search  Database - Public Affairs Index | 1 |
| S8 | TI ( (remote* or virtual*) N3 (ultrason* or (ultra W0 son*) or ultrasound* or (ultra W0 sound*)) ) OR AB ( (remote* or virtual*) N3 (ultrason* or (ultra W0 son*) or ultrasound* or (ultra W0 sound*)) ) | Search modes - Find all my search terms | Interface - EBSCOhost Research Databases  Search Screen - Advanced Search  Database - Public Affairs Index | 0 |
| S7 | TI tele* W0 sonogra* OR AB tele* W0 sonogra* | Search modes - Find all my search terms | Interface - EBSCOhost Research Databases  Search Screen - Advanced Search  Database - Public Affairs Index | 0 |
| S6 | TI ( telesonogra* or (tele W0 sonogra*) ) OR AB ( telesonogra* or (tele W0 sonogra*) ) | Search modes - Find all my search terms | Interface - EBSCOhost Research Databases  Search Screen - Advanced Search  Database - Public Affairs Index | 0 |
| S5 | TI ( (tele* W0 ultrason*) or (tele* W0 ultra W0 son*) or (tele* W0 ultrasound*) or (tele* W0 ultra W0 sound*) or (tele* W0 "US") or (tele* W0 "U/S") ) OR AB ( (tele* W0 ultrason*) or (tele* W0 ultra W0 son*) or (tele* W0 ultrasound*) or (tele* W0 ultra W0 sound*) or (tele* W0 "US") or (tele* W0 "U/S") ) | Search modes - Find all my search terms | Interface - EBSCOhost Research Databases  Search Screen - Advanced Search  Database - Public Affairs Index | 3 |
| S4 | TI ( teleendosonogra* or (tele W0 endosonogra*) or (tele W0 endo W0 sonogra*) or (teleendo W0 sonogra*) ) OR AB ( teleendosonogra* or (tele W0 endosonogra*) or (tele W0 endo W0 sonogra*) or (teleendo W0 sonogra*) ) | Search modes - Find all my search terms | Interface - EBSCOhost Research Databases  Search Screen - Advanced Search  Database - Public Affairs Index | 0 |
| S3 | TI ( teleechogra* or (tele W0 echogra*) or teleechocardiogra* or (tele W0 echocardiogra*) or (tele W0 echo W0 cardiogra*) or (teleecho W0 cardiogra*) or teleechotomogra* or (tele W0 echotomogra*) or (tele W0 echo W0 tomogra*) or (teleecho W0 tomogra*) ) OR AB ( teleechogra* or (tele W0 echogra*) or teleechocardiogra* or (tele W0 echocardiogra*) or (tele W0 echo W0 cardiogra*) or (teleecho W0 cardiogra*) or teleechotomogra* or (tele W0 echotomogra*) or (tele W0 echo W0 tomogra*) or (teleecho W0 tomogra*) ) | Search modes - Find all my search terms | Interface - EBSCOhost Research Databases  Search Screen - Advanced Search  Database - Public Affairs Index | 0 |
| S2 | TI TUS W10 tele* OR AB TUS W10 tele* | Search modes - Find all my search terms | Interface - EBSCOhost Research Databases  Search Screen - Advanced Search  Database - Public Affairs Index | 0 |
| S1 | TI ( teleultrason* or (tele W0 ultrason*) or (tele W0 ultra W0 son*) or (teleultra W0 son*) or teleultrasound* or (tele W0 ultrasound*) or (tele W0 ultra W0 sound*) or (teleultra W0 sound*) or "tele US" or "tele U/S" ) OR AB ( teleultrason* or (tele W0 ultrason*) or (tele W0 ultra W0 son*) or (teleultra W0 son*) or teleultrasound* or (tele W0 ultrasound*) or (tele W0 ultra W0 sound*) or (teleultra W0 sound*) or "tele US" or "tele U/S" ) | Search modes - Find all my search terms | Interface - EBSCOhost Research Databases  Search Screen - Advanced Search  Database - Public Affairs Index | 0 |
