# Supplementary material for: Is remotely supervised ultrasound (tele-ultrasound) inferior to the traditional service model of ultrasound with an in-person imaging specialist? A systematic review
Source: Ultrasound J. 2025 Jul 28;17:34. doi: 10.1186/s13089-025-00440-6 (PMC12304338; doi:10.1186/s13089-025-00440-6)
Supplement: Supplementary file 4 — Supplementary material 4. [file 13089_2025_440_MOESM4_ESM.docx]

**Table 1. Diagnostic Accuracy**

This table summarizes diagnostic performance metrics from included studies, including Proportion of agreement, Sensitivity, Specificity, Positive/Negative predictive values, and interobserver reliability (e.g., ICC)

| **Study** | **Proportion of agreement** | **Sensitivity** | **Specificity** | **Positive**  **predictive value** | **Negative predictive value** | **Interobserver agreement (Intraclass correlation coefficients/Kappa score)** |
| --- | --- | --- | --- | --- | --- | --- |
| Lewin et al. (2006) (6) | 89.6% | NR | NR | NR | NR | NR |
| Mulholland et al. (1999) (7) | 93% | 90.47% | 100% | 100% | 82% | NR |
| McCrossan et al. (2008) (8) | 97% | 96.7% | 96.2% | 98.8% | 89.3% | NR |
| Casey et al. (1996) (9) | 89% | NR | NR | NR | NR | NR |
| Widmer et al. (2003) (10) | 98% | NR | NR | NR | NR | NR |
| Grant et al. (2009) (11) | 96% | 97% | 96% | 98.7% | 88.9% | NR |
| Alsharqi et al. (2022) (12) | Cardiovascular diagnoses   - Aortic valve stenosis: 100% - Aortic valve regurgitation: 100% - Mitral valve stenosis: 100% - Mitral valve regurgitation: 97.25% - Tricuspid valve stenosis: 100% - Tricuspid valve regurgitation: 95.37% - Rheumatic valve disease: 100% - LV enlargement: 97.25% - LVEF:93.58% - LV regional wall motion abnormalities: 96.33% - RV enlargement: 95.41% - RV regional wall motion abnormalities: 99.08% - LA enlargement: 94.5% - RA enlargement: 97.25% - Pericardial effusion: 97.25% - Thrombus: 100% - Tachycardia: 96.33% | NR | NR | NR | NR | Cardiovascular diagnoses (Kappa score)   - Aortic valve stenosis:  k=1 - Aortic valve regurgitation: k =1 - Mitral valve stenosis: k=1 - Mitral valve regurgitation: k=0.921 - Tricuspid valve stenosis: NA - Tricuspid valve regurgitation: k=0.852 - Rheumatic valve disease: k=1 - LV enlargement: k=0.809 - LVEF: k=0.839 - LV regional wall motion abnormalities: k=0.648 - RV enlargement: k=0.423 - RV regional wall motion abnormalities: NA - LA enlargement: k=0.683 - RA enlargement: k=0.386 - Pericardial effusion: k=0.932 - Thrombus: k=1 - Tachycardia: k=0.798 |
| McCrossan et al. (2011) (13) | 97% | 91% | 98% | 91% | 98% | NR |
| Sun et al. (2022) (14) | 93.3% | NR | NR | NR | NR | BI-RAD categories:  0.89 (0.81-0.93)  Ultrasound nodules features:   - Shape 0.62 (0.39-0.77) - Orientation: 1 - Margin: 0.62 (0.43-0.76) - Echo pattern: 0.85 (0.76-0.91) - Posterior features: 0.57 (0.36-0.73) - Calcifications: 0.84 (0.74-0.90) - Vascularity: 0.69 (0.53-0.81) - Internal characteristics: 0.85 (0.75-0.91)   Target nodules measurements:   - Transverse diameter: 0.98 (0.96-0.99) - Anterior-posterior diameter: 0.96 (0.94-0.98) - Longitudinal diameter: 0.93 (0.86-0.96) |
| Li et al. (2022) (15) | 89.4% | 89.4% | 77.4% | 89.4% | 77.4% | ACR TI-RAD categories:  0.791 (0.672-0.870)  Ultrasound nodules features:   - Composition: 0.819 (0.714-0.889) - Echogenicity: 0.694 (0.524-0.806) - Shape: 0.788 (0.668-0.868) - Margin: 0.657 (0.484-0.781) - Echogenic foci: 0.801 (0.686-0.877) - Vascularity: 0.775 (0.649-0.840)   Nodules measurements:   - Transverse diameter: 0.979 (0.965-0.987) - Anterior-posterior diameter: 0.984 (0.9730.990) - Longitudinal diameter: 0.961 (0.935-0.976) |
| Marini et al. (2021) (16) | 98.3% | NR | NR | NR | NR | Lobe diameters:   - Right lobe AP: 0.37 (0.04-0.58) - Right lobe transverse: 0.57 (0.35-0.71) - Left lobe AP: 0.42 (0.02-0.64) - Left lobe transverse: 0.58 (0.01-0.79) - Isthmus lobe AP: 0.48 (-0.22 to 0.77) |
| Marini et al. (2021) (17) | *All exams*   - Liver Echogenicity: 99.3% - Liver Abnormal: 86.1% - Gallbladder: 70.1% - Pancreas Abnormal: 43.4% - Right Kidney Abnormal: 65.2% - Exam Abnormal: 94%   *Ignoring non-visualized cases*   - Liver Echogenicity: 99.3% - Liver Abnormal: 99.2% - Gallbladder: 92.7% - Pancreas Abnormal: 100% - Right Kidney Abnormal: 98.9% - Exam Abnormal: 94% | - Cholelithiasis: 84.2% (60.4 - 96.6%) - Cholelithiasis after consensus read: 89.5% (66.9 - 98.7%) | - Cholelithiasis: 97.7% (91.9 - 99.7%) - Cholelithiasis after consensus read: 97.7% (91.9 - 99.7%) | NR | NR | NR |
| Jemal et al. (2022) (18) | - Placental grading (Grannum classification): 79% - Fetal cardiac activity 98% - Fetal congenital anomaly 98% - Placental location 97% - Intrauterine fetal demise: 100% - Intrauterine growth restriction: 100% - Placenta previa: 100% - Ventriculomegaly: 99% - Anencephaly: 100% - Spina bifida: 99% - Cephalocele: 99% - Fetal hydrops: 98% - Assessment of fetal presentation: 100% - Biophysical profile: 94% - Anatomic assessments: 100% | NR | NR | NR | NR | NR |
| Toscano et al. (2021) (19) | - Confirm live fetus (based on cardiac activity): 76.2% - Fetal number: 100% - Fetal presentation: 95.8% - Placental location 85.6% - Placenta Previa 96% - Placenta Previa (consensus read): 96.8% - Amniotic fluid volume 99.2% - Normal exam 95.2% - Normal exam (consensus read) 96% - Follow-up recommendation (% normal) 99.2% | NR | NR | NR | NR | Fetal biometry  *Second trimester*   - Biparietal diameter 0.84 (0.54-0.96) - Head circumference 0.84 (0.69-0.91) - Abdominal circumference 0.67 (0.45-0.8) - Femur length 0.83 (0.7-0.91) - Estimated gestational age 0.94 (0.65-0.98)   *Third trimester:*   - Biparietal diameter 0.33 (-0.1-0.64) - Head circumference 0.38 (0.06-0.62) - Abdominal circumference 0.28 (0.02-0.52) - Femur length 0.68 (0.32-0.87) - Estimated gestational age 0.64 (-0.02-0.86)   *All exams:*   - Biparietal diameter 0.89 (0.5-0.96) - Head circumference 0.86 (0.71-0.92) - Abdominal circumference 0.81 (0.69-0.88) - Femur length 0.93 (0.88-0.96) - Estimated gestational age 0.95 (0.69-0.98) |
| Evangelista et al. (2016) (20) | NR | Cardiovascular diagnoses   - AS: 98.4% (90.7 to 99.9) - AR:  96.8% (82.0 to 99.8) - MR: 96.0% (85.4 to 99.3) - MS:  100% (31.9 to 100) - TR: 80.9% (66.3 to 90.8) - HCM: 87.5% (44.7 to 99.3) - LV dysf: 90% (75.4 to 96.7) - LVH: 92.5% (86.3 to 96.1) - LA dilation: 62.5% (50.9 to 72.8) - AA dilation: 76% (61.5 to 86.5) | Cardiovascular diagnoses   - AS: 92.1% (88.8 to 93.9) - AR:  98.6% (97.4 to 99.3) - MR:  98.6% (97.3 to 99.3) - MS: 98.9% (94.6 to 99.6) - TR: 98.6% (97.4 to 99.3) - HCM: 99.5% (98.6 to 99.8) - LV dysf: 97.1% (95.5 to 98.1) - LVH: 96.5% (94.7 to 97.8) - LA dilation: 93.9% (91.8 to 96.5) - AA dilation: 97.9% (96.5 to 98.7) | Cardiovascular diagnoses   - AS:  53.7% (44.5 to 62.7 - AR: 75.6% (53.3 to 87.1) - MR: 83.5% (70.5 to 91.1) - MS: 27.3% (17.3 to 60.1) - TR: 77.2% (61.7 to 88.0) - HCM: 63.6% (31.6 to 87.6) - LV dysf: 63.1% (49.3 to 75.2) - LVH: 84.9% (77.8 to 90.1) - LA dilation: 54.4% (43.7 to 64.7) - AA dilation: 71.7% (57.4 to 81.8) | Cardiovascular diagnoses   - AS:  99.8% (99.0 to 99.9) - AR:  99.8% (99.1 to 100) - MR:  99.7% (98.8 to 99.9) - MS:  100% (99.6 to 100) - TR: 98.9% (97.7 to 99.) - HCM:  99.9% (99.1 to 100) - LV dysf: 99.4% (98.4 to 99.8) - LVH: 98.4% (96.9 to 99.1) - LA dilation: 95.6% (93.7 to 96.9) - AA dilation: 98.5% (97.0 to 99.1) | NR |
| Hjorth-Hansen et al. (2020) (21) | NR | 100%/43% | 95%/97% | NR | NR | Echocardiographic indices   - LVEF: 0.78 - LV end-diastolic volume: 0.85 - LV internal end-diastolic diameter: 0.8 - LA end-systolic volume index: 0.75 - Mitral early diastolic velocity: 0.94 - Mitral annular early diastolic velocity: 0.82 - Mitral annular systolic velocity: 0.8 - Mitral E/A ratio: 0.88 - E/e’ ratio: 0.088 - Tricuspid regurgitation peak velocity: 0.71 - LV end-diastolic length: 0.74 - LA end-systolic length: 0.72 - IVS end-diastolic thickness: 0.62 - LV posterior wall end-diastolic thickness: 0.6 - Pleural effusion: 0.88 |
| Kaneko et al. (2021) (22) | NR | NR | NR | NR | NR | Echocardiographic indices   - LVIDd: 0.76 - LVIDs: 0.84 - LVEF: 0.68 - LAD: 0.83 - TAPSE: 0.44 |
| Sable et al. (2002) (23) | NR | NR | NR | NR | NR | NR |
