# Supplementary material for: Is remotely supervised ultrasound (tele-ultrasound) inferior to the traditional service model of ultrasound with an in-person imaging specialist? A systematic review
Source: Ultrasound J. 2025 Jul 28;17:34. doi: 10.1186/s13089-025-00440-6 (PMC12304338; doi:10.1186/s13089-025-00440-6)
Supplement: Supplementary file 5 — Supplementary material 5. [file 13089_2025_440_MOESM5_ESM.docx]

**Table 2. Table of Studies**

| **Study authors, country and purpose** | **Study design, period and setting** | **Patient recruitment** | **Index test 1** | **Index test 2 (if available)** | **Reference test** | **Outcomes** | **Findings** |
| --- | --- | --- | --- | --- | --- | --- | --- |
| **Ultrasound indication: Echocardiography** | | | | | | | |
| Author(s):   - Lewin et al. (2006)   Country(ies):   - Washington, United States | Study design:   - Non-randomized comparative accuracy study   Setting:   - Multi-center   Study period:   - February 2002 to December 2004 (35 months) | Recruitment:   - Total number of patients recruited: 1421 - Excluded based on criteria: 0 - Declined to participate: 0 - Not approached: 0 - Attended: 1421 (1424 echocardiograms)   *Index test 1*   - Number of patients: 766 (769 echocardiograms) - Dropouts/Excluded: 3 - Analyzed: 766 echocardiograms   *Index test 2*   - Number of patients: 655 - Dropouts: 0 - Analyzed: 655   *Reference test*   - Number of patients: 99 - Dropouts: 0 - Analyzed: 99 | **Tele-ultrasound**  Operator(s):   - Local sonographers   Real-time guidance:   - Yes   Mentors:   - Expert   Training:   - Practice - Length: NR   Transmission:   - Real-time   Interpreter(s) of the results:   - A single paediatric cardiologist at CHRMC | **Video-taped echo studies**  Operator(s):   - NR   Interpreter(s) of the results:   - NR | **Subsequent follow-up in the CHRMC cardiology clinics**  Operator(s):   - NR   Interpreter(s) of the results:   - The same cardiologist involved in all tele-echo studies | - The **accuracy** of real-time echocardiography studies conducted via telemedicine and prerecorded video studies (Index test 1 & Reference test and Index test 2 & Reference test) - The **image quality** of tele-ultrasound | **Patient care quality**  Proportion of agreement:  *Index test 1 & Reference test:* 60/67 (89.6%)  *Index test 2 & Reference test:* 20/32 (63%)  Sensitivity: NR  Specificity: NR  Positive Predictive Value (PPV): NR  Negative Predictive Value (NPV): NR  Kappa score: NR  Intraclass Correlation Coefficient (ICC): NR  Change in diagnosis/treatment /referral:  *Index test 1 & Reference test*   - Total discrepancies: 7/67 (10%) - Change in diagnosis: 1/67 (1%) - Minor discrepancies: 6/67 (9%)   *Index test 2 & Reference test*   - Total discrepancies: 12/32 (38%) - Change in diagnosis: 10/32 (31%) - Minor discrepancies: 2/32 (6%)   Patients’ satisfaction rate: NR  **Service quality**  Time to perform ultrasound: NR  Quality of images  *Index test 1*   - 726/769 (94%) were excellent quality - 40/769 (5%) were adequate quality - 3/769 (0.4%) of unsatisfactory quality   Performers’ satisfaction rate: NR  **Access to care**   - NR |
| Author(s):   - Mulholland et al. (1999)   Country(ies):   - UK | Study design:   - Paired comparative accuracy study   Setting:   - Single center   Study period:   - September 1995 to September 1997 (25 months) | Recruitment:   - Total number of patients recruited: 63 - Excluded based on criteria: 0 - Declined to participate: 0 - Not approached: 0 - Attended: 63   *Index test 1*   - Number of patients:  63 - Dropouts: 0 - Analyzed: 61 (2 were inadequate quality for assessment)   *Index test 2*   - Number of patients: 63 - Dropouts: 0 - Analyzed: 63   *Reference test*   - Number of patients: 61 - Dropouts: 0 - Analyzed: 61 | **Tele-echocardiography consulting with paediatric cardiologist**  Operator(s):   - Attending paediatricians   Real-time guidance:   - Yes   Mentors:   - Paediatric cardiologist   Training:   - NR   Transmission:   - Real-time   Interpreter(s) of the results:   - Paediatric cardiologists | **Echocardiography by attending paediatrician**  Operator(s):   - Attending paediatricians   Interpreter(s) of the results:   - Attending paediatricians | **Direct consultation by the paediatric cardiologist and echocardiography**  Operator(s):   - Paediatric cardiologists   Interpreter(s) of the results:   - Paediatric cardiologists   0 | - **Accuracy** of diagnoses from tele-echocardiography and echocardiography by attending paediatricican (Index test 1 & Reference test and Index test 2 & Reference test) | **Patient care quality**  Proportion of agreement  *Index test 1 & Reference test:* 93%  *Index test 2 & Reference test:* 66%  Sensitivity:  *Index test 1 & Reference test:* 90.47%  Specificity:  *Index test 1 & Reference test:* 100%  Positive Predictive Value (PPV): 100%  Negative Predictive Value (NPV): 82%  Kappa score: NR  Intraclass Correlation Coefficient (ICC): NR  Change in diagnosis/treatment /referral:   - 47 infants (74%) avoided transferring (28 with minor congenital heart disease and 19 with no abnormality)   Patients’ satisfaction rate: NR  ***Sub-population:*** NR  **Service quality**  Time to perform ultrasound: NR  Quality of images:   - 61/63 (97%) were diagnostic quality - 2/63 (3%) were not diagnostic quality - 1 poor echocardiographic window due to baby’s skin condition - 1 poor arch view   Performers’ satisfaction rate: NR  **Access to care**  Transportation time: NR  Transportation cost:   - Saved 47 transfers with estimated £300 (US$480) per transfer - Total savings £14,100(US$22,560) - £6,500 (US$10,400 cost of equipment rental, telephone charges) = £7,600 (US$12,160) |
| Author(s):   - McCrossan et al. (2008)   Country(ies):   - United Kingdom | Study design:   - Paired comparative accuracy study (partially)   Setting:   - Multi-center   Study period:   - 1999-2006 | Recruitment:   - Total number of patients recruited: 132 - Excluded based on criteria: 0 - Declined to participate: 0 - Not approached: 0 - Attended: 132   *Index test 1*   - Number of patients:  132 - Dropouts: 0 - Analyzed: 132   *Index test 2*   - Number of patients:  132 - Dropouts: 0 - Analyzed: 132   *Reference test*   - Number of patients: 121 - Dropouts: 0 - Lost to follow-up: 11 - Analyzed: 116 | **Tele-ultrasound interpreted by consultant paedatric cardiologist**  Operator(s):   - Local paediatricians   Real-time guidance:   - Yes   Mentors:   - Consultant paedatric cardiologist   Training:   - NR   Transmission:   - Real-time - Transmission bandwidth: ISDN at 384 kbit/s   Interpreter(s) of the results:   - Consultant paedatric cardiologist | **In-person ultrasound by local paediatrician**  Operator(s):   - Local paediatricians   Ultrasound machine:   - NR   Interpreter(s) of the results:   - Local paediatrician | **Hand-on echocardiogram by paediatric cardiologist**  Operator(s):   - Paediatric cardiologist   Interpreter(s) of the results:   - Paediatric cardiologist | - Diagnostic **accuracy** of tele-ultrasound (Index test 1 & Reference test - The **accuracy** of the initial echocardiogram by the paediatrician (Index test 2 & Reference test) - **Change in transfer decision** (Index test 1 & Index test 2) | **Patient care quality**  Proportion of agreement  *Index test 1 & Reference test:* 112/116 (97%) had CHD  *Index test 2 & Reference test:* 66/116 (57%) had CHD  Diagnostic accuracy: NR  Sensitivity: 96.7%  Specificity: 96.2%  Positive Predictive Value (PPV): 98.8%  Negative Predictive Value (NPV): 89.3%  Kappa score  *Index test 1 & Reference test:* k=0.69 (95% CI: 0.72-1.0)  *Index test 2 & Reference test:* k=0.14 (95% CI: 0.-0.31)  Intraclass Correlation Coefficient (ICC): NR  Change in diagnosis/treatment /referral:  *Index test 1 & Index test 2:*   - 95/132 (72%) transfers were avoided - 15/132 (11.3%) were urgently transferred - 12/132 were electively transferred to regional unit within 84 hours   Patients’ satisfaction rate: NR  **Service quality**   - NR   **Access to care**   - NR |
| Author(s):   - Kaneko et al. (2021)   Country(ies):   - Japan | Study design:   - Paired comparative accuracy study   Setting:   - Singe-center   Study period   - NR | Recruitment:   - Total number of patients recruited: 31 - Excluded based on criteria: 0 - Declined to participate: 0 - Not approached: 0 - Attended: 31   *Index test 1*   - Number of patients: 31 - Dropouts: 0 - Analyzed: 31   *Index test 2*   - Number of patients: 31 - Dropouts: 0 - Analyzed: 31   *Reference test*   - Number of patients: 31 - Dropouts: 0 - Analyzed: 31 | **Tele-ultrasound interpreted by a remote specialist**  Site:   - Remote site: Juntendo University Hospital - Interpretation site: Another building at Juntendo University Hospital   Operator(s):   - A trainee   Real-time guidance:   - Yes   Mentors:   - Remote echocardiography specialist   Training:   - No training   Transmission:   - Real-time - Transmission bandwidth: NR   Interpreter(s) of the results:   - Remote echocardiography specialist | **In-person ultrasound interpreted by the trainee**  Site:   - Juntendo University Hospital   Operator(s):   - A trainee   Experience in ultrasound:   - Operator(s): NR   Ultrasound machine:   - Handheld ultrasound   Interpreter(s) of the results:   - The trainee | **In-person ultrasound by a blinded specialist**  Operator(s):   - A blinded specialist   Interpreter(s) of the results:   - The blinded specialist | - The **accuracy** of examinations under tele-advice (Index test 1 & Reference test - The **accuracy** of the examinations by trainees (Index test 2 & Reference test) | **Patient care quality**  Proportion of agreement: NR  Sensitivity: NR  Specificity: NR  Positive Predictive Value (PPV): NR  Negative Predictive Value (NPV): NR  Kappa score  ***Severity of valvular heart diseases***  *Index test 1 & Reference test*   - Aortic regurgitation: 0.9 - Aortic Stenosis: 0.85 - Mitral regurgitation: 0.85 - Tricuspid regurgitation: 1.00   *Index test 2 & Reference test*   - Aortic regurgitation: 0.38 - Aortic Stenosis: 0.21 - Mitral regurgitation: 0.51 - Tricuspid regurgitation: 0.55   ***Screening cardiac dysfunction***  *Index test 1 & Reference test*   - HFpEF, HFmrEF vs HFrEF: 1.00 - LV regional wall motion abnormality: 0.87 - Valvular heart disease:1.00 - Abnormal TAPSE (<17mm): 0.773   *Index test 2 & Reference test*   - HFpEF, HFmrEF vs HFrEF: 0.318 - LV regional wall motion abnormality: 0.585 - Valvular heart disease: 0.616 - Abnormal TAPSE (<17mm): 0.497   Intraclass Correlation Coefficient (ICC)  *Index test 1 & Reference test*   - LVIDd: 0.76 - LVIDs: 0.84 - LVEF: 0.68 - LAD: 0.83 - TAPSE: 0.44   *Index test 2 & Reference test*   - LVIDd: 0.96 - LVIDs: 0.93 - LVEF: 0.99 - LAD: 0.89 - TAPSE: 0.90   Change in diagnosis/treatment /referral: NR  Patients’ satisfaction rate: NR  **Service quality**   - NR   **Access to care**   - NR |
| Author(s):   - Hjorth‐Hansen et al. (2020)   Country(ies):   - Norway | Study design:   - Paired comparative accuracy study   Setting:   - Single center   Study period:   - October 2016 to February 2017 (4 months) | Recruitment:   - Total number of patients recruited: 50 - Excluded based on criteria: 0 - Declined to participate: 0 - Not approached: 0 - Attended: 50   *Index test 1*   - Number of patients: 50 - Dropouts: 0 - Analyzed: 50   *Index test 2*   - NA   *Reference test*   - Number of patients: 50 - Dropouts: 0 - Analyzed: 50 | **Tele-ultrasound**  Operator(s):   - Registered cardiac nurses   Real-time guidance:   - NR   Mentors:   - NR   Training:   - Lecture and Practice - Length: NR   Transmission:   - Near Real-time   Interpreter(s) of the results:   - The out-of-hospital cardiologist who was blinded to previous echocardiogram and patient’s histories | **NA** | **Echocardiography by in-house physicians experienced in echocardiography**  Operator(s):   - In-house physicians experienced in echocardiography   Interpreter(s) of the results:   - In-house physicians experienced in echocardiography | - The **agreement** of the measurements and HF classification by the telemedical approach and reference test (Index test 1 & Reference test) | **Patient care quality**  Proportion of agreement: NR  Sensitivity:  *For the detection of at least moderate mitral stenosis, mitral regurgitation, and tricuspid regurgitation:* 100%  *For the detection of at least moderate aortic stenosis:* 43%  Specificity:  *For the detection of at least moderate mitral stenosis, mitral regurgitation, and tricuspid regurgitation:* >95%  *For the detection of at least moderate aortic stenosis*: 97%  Kappa score  *Classification of the category of HF*  k = 0.73 (p<0.001)  Correlation (P value)  *Echocardiographic indices*   - LVEF: 0.78 (0.002) - LV end-diastolic volume: 0.85 (<0.001) - LV internal end-diastolic diameter: 0.8 (0.01) - LA end-systolic volume index: 0.75 (0.004) - Mitral early diastolic velocity: 0.94 (<0.001) - Mitral annular early diastolic velocity: 0.82 (<0.001) - Mitral annular systolic velocity: 0.8 (0.001) - Mitral E/A ratio: 0.88 (0.001) - E/e’ ratio: 0.088 (<0.001) - Tricuspid regurgitation peak velocity: 0.71 (0.007) - LV end-diastolic length: 0.74 (0.004) - LA end-systolic length: 0.72 (0.006) - IVS end-diastolic thickness: 0.62 (0.02) - LV posterior wall end-diastolic thickness: 0.6 (0.03) - Pleural effusion: 0.88 (<0.001)   Change in diagnosis/treatment /referral: NR  Patients’ satisfaction rate: NR  **Service quality**  Time to perform ultrasound:   - Time used from the start echocardiography to the finalized report: 1.32 ± 0.36 (1.58) hours - Time used for echocardiographic recordings by nurse: 0.48 ± 0.25 (0.93) hours - Time used for transfer of echocardiograms: 0.36 ± 0.26 (1.20) hours - Time used from echocardiograms uploaded to finalized report by cardiologist: 0.56 ± 0.16 (1.20) hours - Time used for analyses of echocardiograms by cardiologist: 0.20 ± 0.06 (0.27) hours   **Access to care**   - NR |
| Author(s):   - Casey et al. (1996)   Country(ies):   - United Kingdom | Study design:   - Paired comparative accuracy study   Setting:   - Single center   Study period:   - 4 months | Recruitment:   - Total number of patients recruited: 10 - Excluded based on criteria: 0 - Declined to participate: 0 - Not approached: 0 - Attended: 10   *Index test 1*   - Number of patients: 10 - Dropouts: 0 - Analyzed: 9   *Index test 2*   - NA   *Reference test*   - Number of patients: 9 - Dropouts: 0 - Analyzed: 9 | **Tele-echocardiography**  Operator(s):   - Pediatrician   Real-time guidance:   - Yes   Mentors:   - Pediatric cardiologist   Training: NR  Transmission:   - Real-time - Transmission bandwidth: 128 kbit/s   Interpreter(s) of the results:   - Pediatric cardiologist | **In-person echocardiography by attending paediatrician**  Operator(s):   - Attending paediatrician   Experience in ultrasound:   - Operator(s): NR   Ultrasound machine:   - NR   Interpreter(s) of the results:   - Attending paediatrician | **In-person echocardiography by paediatric cardiologist**  Operator(s):   - Paediatric cardiologist   Interpreter(s) of the results:   - Paediatric cardiologist | - The **diagnostic accuracy** tele-echocardiography (Index test 1 & Reference test) | **Patient care quality**  Proportion of agreement:  *Index test 1 & Reference test*   - 8/9 (89%) - 1 could not be diagnosed by tele-echocardiography because of poor echocardiographic image acquisition   Sensitivity: NR  Specificity: NR  Positive Predictive Value (PPV): NR  Negative Predictive Value (NPV): NR  Kappa score: NR  Intraclass Correlation Coefficient (ICC): NR  Change in diagnosis/treatment /referral:   - 8 cases avoided being transferred to the regional referral unit for diagnosis - 1 case required follow-up by paediatric cardiologist   Patients’ satisfaction rate: NR  **Service quality**   - NR   **Access to care**   - NR |
| Author(s):   - Widmer et al. (2003)   Country(ies):   - Switzerland | Study design:   - Paired comparative accuracy study   Setting:   - Singe-center   Study period:   - January 1998 to January 2002 (48 months) | Recruitment:   - Total number of patients recruited: 194 - Excluded based on criteria: 0 - Declined to participate: 0 - Not approached: 0 - Attended: 194   *Index test 1*   - Number of patients: 194 - Dropouts: 0 - Analyzed: 194 patients with 214 tele-echocardiograms   *Index test 2*   - NA   *Reference test*   - Number of patients: 194 - Dropouts: 0 - Analyzed: 194   *Subpopulation*   - Children had subsequent face-to-face echocardiography - Number of patients: 129 | **Tele-ultrasound**  Operator(s):   - Local sonography technician   Real-time guidance:   - Yes   Mentors:   - Paediatric cardiologist   Training:   - Lecture and Practice - Length: 2 years   Transmission:   - Real-time and video-taped - Transmission bandwidth: telemedicine link across three ISDN lines with a total transmission rate of 384 kbit/s   Interpreter(s) of the results:   - Remote echocardiography specialist | **NA** | **Paediatric cardiologist’s interpretation**  Number of References: 3   - Echocardiography videotapes were reviewed by the paediatric cardiologist if tele-echocardiography findings were normal - Re-examinations by the paediatric cardiologist in inconclusive cases - Face-to-face consultation and echocardiographic follow-up   Operator(s):   - Local experienced sonography technician acquired echocardiography videotape - Paediatric cardiologist acquired in-person or re-examining echocardiography   Interpreter(s) of the results:   - Paediatric cardiologist | - The diagnostic accuracy of tele-echocardiography (Index test 1 & Reference test) | **Patient care quality**  Proportion of agreement   - 191/194 (98%) patients had correct diagnosis - 3/194 (2%) patients had uncertain or incorrect diagnosis   Sensitivity: NR  Specificity: NR  Positive Predictive Value (PPV): NR  Negative Predictive Value (NPV): NR  Kappa score: NR  Intraclass Correlation Coefficient (ICC): NR  Change in diagnosis/treatment /referral:   - 6 cases avoided urgent transfer   Patients’ satisfaction rate: NR  **Service quality**  Time to perform ultrasound: NR  Image quality:   - All were sufficient for interpretation except for one patient with distally located coarctation   Performers’ satisfaction rate or confidence level: NR  **Access to care**   - NR |
| Author(s):   - Evangelista et al. (2016)   Country(ies):   - Spain | Study design:   - Paired comparative accuracy study   Setting:   - Multi-center   Study period:   - 6 months | Recruitment:   - Total number of patients recruited: 1312 - Excluded based on criteria: 0 - Declined to participate: 0 - Not approached: 0 - Attended: 1312   *Index test 1*   - Number of patients: 1312 - Dropouts: 0 - Analyzed: 1312   *Index test 2*   - Number of patients: 1312 - Dropouts: 0 - Analyzed: 1312   *Reference test*   - Number of patients: 859 - Dropouts: 85 - Analyzed: 774   Subpopulation:   - NR | **HCU interpreted by remote experts**  Operator(s):   - FDs   Real-time guidance:   - No   Mentors:   - NA   Training:   - Lecture and Practice - Length: 7 hours per day for 4 days   Transmission:   - Storage - Transmission bandwidth: Broadband internet connection   Interpreter(s) of the results:   - Remote experts | **HCU interpreted by FDs**  Operator(s):   - FDs   Interpreter(s) of the ultrasound:   - FDs | **Conventional echocardiographic (CE) studies**  Operator(s):   - Blinded independent expert echocardiographers   Interpreter(s) of the results:   - Same blinded independent expert echocardiographers | - FDs and remote expert diagnosis concordance (Index test 1 & Index test 2) - Agreement and accuracy of HCU diagnosis by remote experts compared with CE (Index test 1 & Reference test) - Changes in FDs’ management after remote experts’ interpretation (Index test 1 & Index test 2) | **Patient care quality**  Proportion of agreement: NR  Sensitivity:  *Index test 1 & Index test 2:* ranging from 41.4-72.7%   - AS: 50.0 (0.39-0.63) - AR: 58.3 (43.3 to 73.3) - MR: 72.7 (61.2 to 84.2) - MS: 62.8 (22.7 to 100) - TR: 41.4 (21.7 to 61.0) - HCM:  44.4 (6.4 to 82.5) - LV dysf: 50 (30.4 to 69.6) - LVH: 71.4 (63.1 to 79.7) - LA dilation: 41.5 (25.2 to 57.8) - AA dilation: 54.1 (37.1 to 70.2)   *Index test 1 & Reference test:* ranging from 62.5-100%   - AS: 98.4 (90.7 to 99.9) - AR:  96.8 (82.0 to 99.8) - MR: 96.0 (85.4 to 99.3) - MS:  100 (31.9 to 100) - TR: 80.9 (66.3 to 90.8) - HCM: 87.5 (44.7 to 99.3) - LV dysf: 90 (75.4 to 96.7) - LVH: 92.5 (86.3 to 96.1) - LA dilation: 62.5 (50.9 to 72.8) - AA dilation:  76 (61.5 to 86.5)   Specificity:  *Index test 1 & Index test 2:* ranging from 92.7-99.8%   - AS: 98.1 (97.0 to 99.1) - AR: 99.0 (98.3 to 99.6) - MR: 97.7 (96.8 to 98.6) - MS: 98.1 (97.3 to 98.9) - TR: 98.9 (98.3 to 99.5) - HCM: 99.8 (99.6 to 100) - LV dysf: 92.7 (91.3 to 94.2) - LVH: 97.4 (96.7 to 98.6) - LA dilation: 97.7 (96.8 to 98.6) - AA dilation: 99.1 (98.4 to 99.6)   *Index test 1 & Reference test:* ranging from 92.1-99.5%   - AS: 92.1 (88.8 to 93.9) - AR:  98.6 (97.4 to 99.3) - MR:  98.6 (97.3 to 99.3) - MS: 98.9 (94.6 to 99.6) - TR: 98.6 (97.4 to 99.3) - HCM: 99.5 (98.6 to 99.8) - LV dysf: 97.1 (95.5 to 98.1) - LVH: 96.5 (94.7 to 97.8) - LA dilation: 93.9 (91.8 to 96.5) - AA dilation: 97.9 (96.5 to 98.7)   Positive Predictive Value (PPV):  *Index test 1 & Index test 2:* ranging from 13.9-74.4%   - AS: 49.2 (35.3 to 63.0) - AR: 68.3 (52.8 to 83.8) - MR: 62.3 (50.9 to 73.8) - MS: 18.7 (1.9 to 31.7) - TR: 46.2 (25.1 to 67.2) - HCM: 66.7 (20.6 to 100) - LV dysf: 13.9 (6.9 to 20.8) - LVH: 74.4 (66.2 to 82.6) - LA dilation: 37.0 (21.9 to 52.0) - AA dilation: 64.5 (45.4 to 80.2)   *Index test 1 & Reference test:* ranging from 27.3-84.9%   - AS:  53.7 (44.5 to 62.7 - AR: 75.6 (53.3 to 87.1) - MR: 83.5 (70.5 to 91.1) - MS: 27.3 (17.3 to 60.1) - TR: 77.2 (61.7 to 88.0) - HCM: 63.6 (31.6 to 87.6) - LV dysf: 63.1 (49.3 to 75.2) - LVH: 84.9 (77.8 to 90.1) - LA dilation: 54.4 (43.7 to 64.7) - AA dilation: 71.7 (57.4 to 81.8)   Negative Predictive Value (NPV):  *Index test 1 & Index test 2:* ranging from 97-99.8%   - AS:  98.1 (97.2 to 99.0) - AR:  98.4 (97.7 to 99.1) - MR:  98.5 (97.8 to 99.3) - MS: 99.8 (99.5 to 100) - TR: 98.7 (98.0 to 99.3) - HCM: 99.6 (99.2 to 99.9) - LV dysf: 98.7 (98.1 to 99.4) - LVH: 97.0 (96.0 to 98.0) - LA dilation: 98.1 (97.3 to 98.9) - AA dilation:  98.7 (97.8 to 99.2)   *Index test 1 & Reference test:* ranging from 95.6-100%   - AS:  99.8 (99.0 to 99.9) - AR:  99.8 (99.1 to 100) - MR:  99.7 (98.8 to 99.9) - MS:  100 (99.6 to 100) - TR: 98.9 (97.7 to 99.) - HCM:  99.9 (99.1 to 100) - LV dysf:  99.4 (98.4 to 99.8) - LVH: 98.4 (96.9 to 99.1) - LA dilation:  95.6 (93.7 to 96.9) - AA dilation: 98.5 (97.0 to 99.1)   Kappa score  *Index test 1 & Index test 2*   - AS:  0.53 (0.39–0.63) - AR: 0.61 (0.50 to 0.74) - MR: 0.65 (0.56 to 0.74) - MS: 0.29 (0.9 to 0.47) - TR: 0.42 (0.25 to 0.59) - HCM:  0.53 (0.23 to 0.83) - LV dysf:  0.51 (0.37 to 0.62) - LVH: 0.70 (0.60 to 0.78) - LA dilation:  0.38 (0.24 to 0.50) - AA dilation:  0.54 (0.43 to 0.71)   *Index test 1 (Group A) & Reference test*   - AS: 0.66 (0.57 to 0.74) - AR:  0.84 (0.75 to 0.93) - MR: 0.88 (0.81 to 0.94) - MS:  0.43 (0.13 to 0.82) - TR: 0.78 (0.68 to 0.88) - HCM:  0.73 (0.50 to 0.96) - LV dysf:  0.72 (0.62 to 0.83) - LVH: 0.77 (0.67 to 0.88) - LA dillation:  0.63 (0.53 to 0.73) - AA dilation:  0.71 (0.61 to 0.0.82)   Intraclass Correlation Coefficient (ICC): NR  Change in diagnosis/treatment /referral:  *Index test 1 & Index test 2*   - Requested conventional echocardiography: 647/859 (859-212) (75%) - Referred to Cardiology: 25/41 (41-16) (61%) - Clinical follow-up: 105/247 (247-142) (42%) - Discharge: 80/165 (165-85) (48%)   Patients’ satisfaction rate: NR  **Service quality:**  Time to perform: ultrasound: NR  Image quality:  *Index test 1*   - Good: 35.4% - Acceptable: 45.4% - Poor: 19.2% - Inconclusive: 8.7%   Performer’s satisfaction rate or confidence level: NR  **Access to care:**   - NR |
| Author(s):   - Sable et al. (2002)   Country(ies):   - USA | Study design:   - Paired comparative accuracy study   Setting:   - Multi-center   Study period:   - April 1998 to October 2000 (30 months) | Recruitment:   - Total number of patients recruited: 364 - Excluded based on criteria: 0 - Declined to participate: 0 - Not approached: 0 - Attended: 364   *Index test 1*   - Number of patients: 364 - Dropouts: 0 - Analyzed: 364 patients with 500 telemedicine transmissions   *Index test 2*   - NA   *Reference test*   - Number of patients: 364 - Dropouts: 0 - Analyzed: 364 | **Tele-ultrasound**  Operator(s):   - Sonographers   Real-time guidance:   - Yes   Mentors:   - Pediatric cardiologists licensed in both the District of Columbia and Maryland   Training:   - Lecture and Practice - Length: NR   Transmission:   - Real-time and Storage - Transmission bandwidth: 3 ISDN (384 kilobits per second)   Interpreter(s) of the results:   - Pediatric imaging cardiologists | **NA** | **Interpretation on video-taped echocardiogram or Subsequent follow-up echocardiogram**  Number of reference tests: 2  Operator(s):   - Sonographers   Interpreter(s) of the results:   - Physician who was covering the echocardiography laboratory when the tape arrived | - The **agreement** between remote-mentored echocardiography and the subsequent review (Index test 1 & Reference test) - **Examination time** of tele-echocardiography | **Patient care quality**  Proportion of agreement: NR  Sensitivity: NR  Specificity: NR  Positive Predictive Value (PPV): NR  Negative Predictive Value (NPV): NR  Kappa score: NR  Intraclass Correlation Coefficient (ICC): NR  Change in diagnosis/treatment /referral:  *Diagnosis*   - 1 diagnostic change after videotape interpretation - 3/264 diagnostic changes after subsequent follow-up   *Treatment*   - 151/500 (30%) studies had altered immediate patient management - 76/151 (50%) had indomethacin treatment for PDA - 45/151 (30%) had retraction of umbilical venous catheters from the left atrium - 19/151 (13%) had inotropic or anticongestive therapy - 8/151 (5%) had prostaglandin infusion   *Transfer/Transportation*   - 19/364 (5%) were transported to central hospital - 14/364 (4%) avoided transportation   Patients’ satisfaction rate: NR  **Service quality**  Time to perform ultrasound   - Time from request for echocardiogram to completion of the videoconference: 28 ± 14 minutes - Total video conference time: 20 ± 8 minutes - Waiting time: 8 ± 11 minutes - Time to send videotape: 12 ± 16 hours   Quality of images: NR  Performers’ satisfaction rate: NR  **Access to care**  Transportation time:   - Average time saving for cardiologist: 4.2 person-hours/week |
| Author(s):   - Grant et al. (2009)   Country(ies):   - UK | Study design:   - Paired comparative accuracy study   Setting:   - Multicenter   Study period:   - 1999 to 2006 | Recruitment:   - Total number of patients recruited: 124 - Excluded based on criteria: 0 - Declined to participate: 0 - Not approached: 0 - Attended: 124   *Index test 1*   - Number of patients:  124 - Dropouts: 0 - Analyzed: 124   *Index test 2:*   - Number of patients: 124 - Dropouts: 0 - Analyzed: 124   *Reference test:*   - Number of patients: 114 - Dropouts: 5 - Analyzed: - For diagnostic accuracy: 109 - For patient transfers: 124 | **Tele-ultrasound**  Operator(s):   - Attending paediatrician   Real-time guidance:   - Yes   Mentors:   - Paediatric cardiologist   Training:   - NR   Transmission:   - Real-time - Transmission bandwidth: NR   Interpreter(s) of the results:   - An agreement between local pediatricians and pediatric cardiologist at the RPCU | **On-site Echocardiogram**  Operator(s):   - Attending paediatrician   Interpreter(s) of the results:   - Attending paediatrician | **Hands-on consultation and echocardiogram**  Operator(s):   - Paediatric cardiologist at RPCU or at DGHs   Interpreter(s) of the results:   - Paediatric cardiologist at RPCU or DGHs | - **Diagnostic agreement** of tele-ultrasound, on-site echocardiogram, and hands-on echocardiogram (Index test 1 & Reference test and Index test 2 & Reference test) | **Patient care quality**  Proportion of agreement  *Index test 1 & Reference test:* 105/109 (96%) cases were accurately diagnosed  *Index test 2 & Reference test:* 63/109 (58%) cases were accurately diagnosed  Sensitivity: NR  *Index test 1 & Reference test:* 97%  *Index test 2 & Reference test:* 56%  Specificity: NR  *Index test 1 & Reference test:* 96%  *Index test 2 & Reference test:* 64%  Positive Predictive Value (PPV):  *Index test 1 & Reference test:* 98.7%  *Index test 2 & Reference test:* 83.9%  Negative Predictive Value (NPV):  *Index test 1 & Reference test:* 88.9%  *Index test 2 & Reference test:* 30.2%  Kappa score (95% confidence interval)  *Index test 1 & Reference test:* 0.89 (0.71-1.0)  *Index test 2 & Reference test:* 0.14 (0-0.31)  Intraclass Correlation Coefficient (ICC): NR  Change in diagnosis/treatment /referral:   - 93/124 (75%) patient transfers were avoided - 17 patients were urgently transferred - 14 patients were semi-urgently transferred within 48h   Patients’ satisfaction rate: NR  **Service quality**  Time to perform ultrasound  *Index test 1*   - 19.8 min (range 9 - 44 min)   Quality of images: NR  Performers’ satisfaction rate ( 5-point Likert scale):   - Telemedicine is useful: 4.5 ± 0.82 - They felt reassured by the facility: 4.2 ± 1.09   **Access to care**  Cost saved per patient with telemedicine service for each district hospital   - ALT: £1822 - CAH: £608 - AAH: £739 |
| Author(s):   - Alsharqi et al. (2022)   Country(ies):   - India +  UK | Study design:   - Paired comparative accuracy study   Setting:   - Multicenter   Study period:   - February 2019 to July 2021 (29 months) | Recruitment:   - Total  number of patients recruited: 301 - Excluded based on criteria: 0 - Declined to participate: 0 - Attended: 301   *Index test 1*   - Number of patients: 301 - Dropouts: 0 - Analyzed: 109   *Index test 2*   - NA   *Reference test*   - Number of patients: 36 - Dropouts: 0 - Analyzed: 36 | **Tele focused echocardiography**  Operator(s):   - Trained obstetricians   Real-time guidance:   - No   Mentors:   - NA   Training:   - Lecture and Practice - Length: 2 days of lecture and multiple practice sessions   Transmission:   - Storage - Transmission bandwidth: NR   Interpreter(s) of the results:   - Two experts | **NA** | **Standard echocardiogram**  Operator(s):   - NR   Interpreter(s) of the results:   - NR | - The **diagnostic accuracy** of tele-FOCUS (Index test 1 & Reference test) - **Agreement** between two experts who read images of tele-FOCUS (Index test 1) - The ability of tele- FOCUS to detect echocardiographic abnormalities  on scans in which the parameter could be assessed (Index test 1) | **Patient care quality**  Proportion of agreement: ranging from 93.58% to 100%   - Aortic valve stenosis: 100% - Aortic valve regurgitation: 100% - Mitral valve stenosis: 100% - Mitral valve regurgitation: 97.25% - Tricuspid valve stenosis: 100% - Tricuspid valve regurgitation: 95.37% - Rheumatic valve disease: 100% - LV enlargement: 97.25% - LVEF:93.58% - LV regional wall motion abnormalities: 96.33% - RV enlargement: 95.41% - RV regional wall motion abnormalities: 99.08% - LA enlargement: 94.5% - RA enlargement: 97.25% - Pericardial effusion: 97.25% - Thrombus: 100% - Tachycardia: 96.33%   Sensitivity: NR  Specificity: NR  Positive Predictive Value (PPV)  Negative Predictive Value (NPV)  Kappa score  *Index test 1 (Between the experts)*   - Aortic valve stenosis:  k=1 - Aortic valve regurgitation: k =1 - Mitral valve stenosis: k=1 - Mitral valve regurgitation: k=0.921 - Tricuspid valve stenosis: NA - Tricuspid valve regurgitation: k=0.852 - Rheumatic valve disease: k=1 - LV enlargement: k=0.809 - LVEF: k=0.839 - LV regional wall motion abnormalities: k=0.648 - RV enlargement: k=0.423 - RV regional wall motion abnormalities: NA - LA enlargement: k=0.683 - RA enlargement: k=0.386 - Pericardial effusion: k=0.932 - Thrombus: k=1 - Tachycardia: k=0.798   *Index test 1 & Reference test*: NR  Intraclass Correlation Coefficient (ICC): NR  Change in diagnosis/treatment /referral:   - No patients required additional medication or change in delivery plan   Patients’ satisfaction rate: NR  **Service quality**  Time to perform ultrasound: NR  Quality of images: NR  Performers’ satisfaction rate: NR  **Access to care**   - NR |
| **Ultrasound indication: Fetal echocardiography** | | | | | | | |
| Author(s):   - McCrossan et al. (2011)   Country(ies):   - UK | Study design:   - Paired comparative accuracy study   Setting:   - Single center   Study period:   - 20 months | Recruitment:   - Total number of patients recruited: 67 - Excluded based on criteria: 0 - Declined to participate: 0 - Not approached: 0 - Attended: 67   *Index test 1*   - Number of patients: 67 - Dropouts: 0 - Analyzed: 69 (including one set of twin and a repeated tele-echocardiography)   *Index test 2*   - Number of patients: 67 - Dropouts: 0 - Analyzed: 69 including one set of twin and a repeated tele-echocardiography   *Reference test*   - Number of patients: 66 - Dropouts: 1 - Analyzed: 67 (with one set of twin) | **Tele-ultrasound**  Operator(s):   - Sonographer   Real-time guidance:   - Yes   Mentors:   - Remote fetal cardiologist at the regional center   Training:   - NR   Transmission:   - Real-time - Transmission bandwidth: NR   Interpreter(s) of the results:   - Remote fetal cardiologist at the regional center | **In-person FE at a district general hospital (DGH)**  Operator(s):   - Radiographer   Interpreter(s) of the results:   - Radiographer | **In-person FE at regional fetal cardiology unit**  Operator(s):   - Fetal cardiologist   Interpreter(s) of the results:   - Fetal cardiologist | - **Diagnostic accuracy** of remote fetal-tele echocardiograms (Index test 1 & Reference test) - **Performers’ opinions** on fetal tele-echocardiogram - **Quality** of remote fetal tele-echocardiograms (Index test 1) | **Patient care quality**  Proportion of agreement:  *Index test 1 & Reference test:* 97%  *Index test 2 & Reference test:* 68%  Sensitivity  *Index test 1 & Reference test:* 91%  *Index test 2 & Reference test:* 72%  Specificity  *Index test 1 & Reference test:* 98%  *Index test 2 & Reference test*: 67%  Positive Predictive Value (PPV)  *Index test 1 & Reference test:* 91%  *Index test 2 & Reference test:* 28.5%  Negative Predictive Value (NPV)  *Index test 1 & Reference test:* 98%  *Index test 2 & Reference test*: 92.7%  Kappa score  *Index test 1 & Reference test:* k=0.89  *Index test 2 & Reference test:* k=0.25  Intraclass Correlation Coefficient (ICC): NR  Change in diagnosis/treatment /referral: NR  Patients’ satisfaction rate: NR  **Service quality**  Time to perform ultrasound: NR  Quality of images:  *Index test 1*   - Median video quality (IQR) 4/5 (3.5-4.5) - Median audio quality (IQR) 4/5 (3.5-4.5) - Median ease of use (IQR) 4/5  (4-4.5) - Median overall quality (IQR) 4/5  (3.6-4.5)   Performers’ satisfaction rate at the start and end of the study (Likert score 1 – 5)  *Confidence in FE technique*   - Start 2/5 - End 3.8/5   *Confidence in detecting CHD*   - Start 1.8/5 - End 4/5   *Telemedicine equipment was easy to use*   - Start 3.4/5 - End 4.8/5   *Feedback from experts via tele-linked scan is beneficial*   - Start 4.2/5 - End 4.8/5   *Felt awkward performing FE in front of paediatric cardiologist*   - Start 2.6/5 - End 1.6/5   *Saved videos for interpretation were better than a “live telemedicine consultation*   - Start 2/5 - End 1.4/5   **Access to care**   - NR |
| **Ultrasound indication: Obstetrical ultrasound** | | | | | | | |
| Author(s):   - Jemal et al. (2022)   Country(ies):   - Ethiopia | Study design:   - Paired comparative accuracy study   Setting:   - Single center   Study period:   - July 1^st^, 2021 to August 30^th^, 2021 (2 months) | Recruitment:   - Total number of patients recruited: 2795 - Excluded based on criteria: 0 - Declined to participate: 0 - Not approached: 0 - Attended: 2795   *Index test 1*   - Number of patients:  2795 - Dropouts: 0 - Analyzed: 100   *Index test 2*   - NA   *Reference test*   - Number of patients: 2795 - Dropouts: 0 - Analyzed: 100   *Subpopulation:*   - Number of patients: 180 random participants surveyed for experience - Number of patients: 100 random participants surveyed for clinic experience on Saturday | **Tele-ultrasound**  Operator(s):   - HCPs   Real-time guidance:   - Yes   Mentors:   - NR   Training:   - Lecture and Practice - Length: NR   Transmission:   - Real-time and store-and-forward - Transmission bandwidth: NR   Interpreter(s) of the results:   - HCPs | **NA** | **Obstetricians’ interpretation on images acquired by HCPs**  Operator(s):   - HCPs   Interpreter(s) of the results:   - Obstetricians | - **Concordance in interpretations** between HCPs and obstetricians (Index test 1 & Reference test) - **Patient’s experience** with and attitudes towards tele-ultrasound and access to antenatal care - **HCP’s experience** with and attitudes towards tele-ultrasound | **Patient care quality**  Proportion of agreement: ranging from 79-100%   - Placental grading (Grannum classification): 79% - Fetal cardiac activity 98% - Fetal congenital anomaly 98% - Placental location 97% - Intrauterine fetal demise: 100% - Intrauterine growth restriction: 100% - Placenta previa: 100% - Ventriculomegaly: 99% - Anencephaly: 100% - Spina bifida: 99% - Cephalocele: 99% - Fetal hydrops: 98% - Assessment of fetal presentation: 100% - Biophysical profile: 94% - Anatomic assessments: 100%   Sensitivity: NR  Specificity: NR  Positive Predictive Value (PPV): NR  Negative Predictive Value (NPV): NR  Kappa score: NR  Intraclass Correlation Coefficient (ICC): NR  Change in diagnosis/treatment /referral: NR  *Referral*   - 108/2795 (58 from Hambiso Health Centre and 50 from Fitche 1 Health Centre) - Multi gestation: 35 - Malpresentation: 29 - Missed/Incomplete abortion or intrauterine fetal demise: 18 - Ventriculomegaly: 8 - Anencephaly: 5 - Spina bifida: 2 - Oligohydramnios: 2 - Fetal hydrops: 1 - Ovarian cyst: 2   Patients’ satisfaction rate:   - 173/180 (96%) felt comfortable during the procedure - 179/180 (99%) agreed that they would recommend antenatal tele-ultrasound to friends and family - 176/180 (98%) were willing to undergo another antenatal ultrasound through telemedicine - 130/180 (72%) were satisfied with the picture quality - 139/180 (77%) were satisfied with the sound quality - 64/180 (36%) were not comfortable communicating with remote obstetrician - 177/180 (98%) agreed that the encounter was private and confidential - 89/180 (49%) disagreed that they had to wait long to receive healthcare - 54/180 (30%) were unsure or agreed that they had to wait long - 137/180 (76%) agreed that they were given enough information to prepare for the ultrasound - 113/180 (63%) agreed that they had enough time to think about questions and to ask the remote obstetrician   **Service quality**  Time to perform ultrasound: NR  Image quality: NR  Performers’ satisfaction rate or confidence level:   - 100% agreed that they received adequate training for image acquisition - 100% felt confident in their ability to acquire images - 100% enjoyed using telemedicine system - 100% felt that their patients was satisfied with the received level of care - 100% agreed telemedicine is an acceptable method of providing healthcare services - 100% agreed telemedicine improves access to needed healthcare services   **Access to care**  Transportation cost:   - Traveling to health centers: 11.7 ± 12.7 Ethiopian Birr - Traveling to SIUCSH: 20.8 ± 20.9 Ethiopian Birr   Transportation time:   - Traveling to health centers: 44.1 ± 56.7 minutes - Traveling to SIUCSH: 54.2 ± 65.3 minutes   Waiting time: NR |
| Author(s):   - Toscano et al. (2021)   Country(ies):   - Peru + United States | Study design:   - Paired comparative accuracy study   Setting:   - Single center   Study period:   - June 2018 to March 2019 (10 months) | Recruitment:   - Total number of patients recruited: 126 - Excluded based on criteria: 0 - Declined to participate: 0 - Not approached: 0 - Attended: 126   *Index test 1*   - Number of patients: 126 - Dropouts: 0 - Analyzed: 126   *Index test 2*   - NA   *Reference test*   - Number of patients: 126 - Dropouts: 0 - Analyzed: 126 | **Tele-ultrasound (Volume sweep imaging – VSI)**  Operator(s):   - A nurse and a care technician   Real-time guidance:   - No   Mentors:   - NA   Training:   - Lecture and Practice - Length: 4 hours of didactic sessions and 4 hours of hands-on training   Transmission:   - Storage - Transmission bandwidth: NR   Interpreter(s) of the results:   - a Maternal-Fetal Medicine fellow | **NA** | **Standard of care ultrasound**  Operator(s):   - Radiologists   Interpreter(s) of the results:   - Radiologists | - **Overall agreement** and Cohen’s kappa on agreement on categorical variables between VSI and standard of care (SOC) ultrasound (Index test 1 & Reference test) - ICC values were calculated for biometry measurements acquired by VSI and SOC ultrasound | **Patient care quality**  Proportion of agreement: ranging from 76.2-100%   - Confirm live fetus (based on cardiac activity): 76.2% - Fetal number: 100% - Fetal presentation: 95.8% - Placental location 85.6% - Placenta Previa 96% - Placenta Previa (consensus read): 96.8% - Amniotic fluid volume 99.2% - Normal exam 95.2% - Normal exam (consensus read) 96% - Follow-up recommendation (% normal) 99.2%   Sensitivity: NR  Specificity: NR  Positive Predictive Value (PPV): NR  Negative Predictive Value (NPV): NR  Kappa score   - Confirm live fetus (based on cardiac activity): κ not defined - Fetal number: κ not defined - Fetal presentation: κ = 0.78 (0.53-1.0; p< 0.0001) - Placental location κ = 0.74 (0.63-0.85, p< 0.0001) - Placenta Previa: κ not defined - Placenta Previa (consensus read): κ not defined - Amniotic fluid volume: κ not defined - Normal exam: κ = 0.55 (0.2-0.9, p<0.0001) - Normal exam (consensus read): κ = 0.6 (0.25-0.94, p<0.0001) - Follow-up recommendation (% normal): κ not defined   Intraclass Correlation Coefficient (ICC) (Fetal biometry):  *Second trimester exams*   - Biparietal diameter 0.84 (0.54-0.96, p<0.0001) - Head circumference 0.84 (0.69-0.91, p<0.0001) - Abdominal circumference 0.67 (0.45-0.8, p<0.0001) - Femur length 0.83 (0.7-0.91, p<0.0001) - Estimated gestational age 0.94 (0.65-0.98, p<0.0001)   *Third trimester exams*   - Biparietal diameter 0.33 (-0.1-0.64, p< 0.0001) - Head circumference 0.38 (0.06-0.62, p=0.001) - Abdominal circumference 0.28 (0.02-0.52, p=0.015) - Femur length 0.68 (0.32-0.87, p<0.0001) - Estimated gestational age 0.64 (-0.02-0.86, p<0.0001)   *All exams*   - Biparietal diameter 0.89 (0.5-0.96, p<0.0001) - Head circumference 0.86 (0.71-0.92, p<0.0001) - Abdominal circumference 0.81 (0.69-0.88, p<0.0001) - Femur length 0.93 (0.88-0.96, p<0.0001) - Estimated gestational age 0.95 (0.69-0.98, p<0.001)   Change in diagnosis/treatment /referral: NR  Patients’ satisfaction rate: NR  **Service quality:**  Time to perform ultrasound: NR  Image quality:  *Index test 1*   - Excellent (61.1%) - Acceptable (38.1%) - Poor (0.8%)   Performer’s satisfaction rate or confidence level  *Confidence level of readers (3-point Likert scale)*   - Live Fetus 3 (1-3) - Number of Fetuses 3 (1-3) - Fetal presentation 3 (1-3) - Placenta Previa 3 (1-3) - Amniotic Fluid Volume 3 (1-3) - Normal exam 3 (1-3) - Follow-up Recommendation (% normal) 3 (1-3)   **Access to care:**   - NR |
| **Ultrasound indication: Breast Ultrasound** | | | | | | | |
| Author(s):   - Sun et al. (2022)   Country(ies):   - China | Study design:   - Paired comparative accuracy study   Setting:   - Single center   Study period:   - April 2020 to June 2020 (3 months) | Recruitment:   - Total number of patients recruited: 100 - Excluded based on criteria: 1 - Declined to participate: 0 - Not approached: 0 - Attended: 99   *Index test 1*   - Number of patients:  99 - Dropouts: 0 - Analyzed: 99   *Index test 2*   - Number of patients: 99 - Dropouts: 0 - Analyzed: 99   *Reference test*   - Number of patients: 99 - Dropouts: 0 - Analyzed: 99 | **Tele-ultrasound**  Operator(s):   - Trainee B (TB)   Real-time guidance:   - Yes   Mentors:   - Expert in breast US   Training:   - Lecture and Practice - Length: 5 hours   Transmission:   - Real-time - Transmission bandwidth: high-speed network   Interpreter(s) of the results:   - Resident B and the remote expert through discussion | **In-person ultrasound by naive operator**  Operator(s):   - Trainee A (TA)   Interpreter(s) of the results:   - Resident A | **In-person ultrasound and interpretations by experts**  Number of References: 2   - The expert that guided resident B performed the on-site ultrasound and made independent interpretation - 2 other experts experienced US off-site experts were designated to analyze all data acquired by TA and TB in a blind manner   Operator(s):   - The expert who guided Resident B   Interpreter(s) of the results:   - The expert who guided Resident B - 2 other experts experienced US | - **Inter-operator consistency between the two residents and the on-site US expert** was compared (Index test & Reference test + Index 2 & Reference test) - The tele-US and normal US image quality - The target nodule image quality - Comprehensive assessment on a scale of 1-5 | **Patient care quality**  Proportion of agreement:  *Index test 1 & Reference* *test:* 56/60 (93.3%)  *Index test 2 & Reference test:* 38/60 (63.3%)  Sensitivity: NR  Specificity: NR  Positive Predictive Value (PPV): NR  Negative Predictive Value (NPV): NR  Intraclass Correlation Coefficient (ICC) (Interobserver agreement) with two-way random effect model  *Index test 1 & Reference test:*  BI-RADS categories: 0.89 (0.81-0.93)  Ultrasound features:   - Shape 0.62 (0.39-0.77) - Orientation: 1 - Margin: 0.62 (0.43-0.76) - Echo pattern: 0.85 (0.76-0.91) - Posterior features: 0.57 (0.36-0.73) - Calcifications: 0.84 (0.74-0.90) - Vascularity: 0.69 (0.53-0.81) - Internal characteristics: 0.85 (0.75-0.91)   Target nodule measurement:   - Transverse diameter: 0.98 (0.96-0.99) - Anterior-posterior diameter: 0.96 (0.94-0.98) - Longitudinal diameter: 0.93 (0.86-0.96)   *Index test 2 & Reference test:*  BI-RADS categories: 0.73 (0.54-0.85)  Ultrasound features:   - Shape 0.66 (0.43-0.81) - Orientation: NA - Margin: 0.32 (-0.08-0   58)   - Echo pattern: 0.65 (0.43-0.80) - Posterior features: 0.47 (0.17-0.69) - Calcifications: 0.81 (0.66-0.90) - Vascularity: 0.28 (-0.02-0.53) - Internal characteristics: 0.37 (0.08-0.61)   Target nodule measurement:   - Transverse diameter: 0.89 (0.79-0.94) - Anterior-posterior diameter: 0.89 (0.79-0.94) - Longitudinal diameter: 0.89 (0.78-0.94)   Change in diagnosis/treatment /referral: NR  Patients’ satisfaction rate (n=99)  *Tele-US acceptance*   - Yes: 63 (63.6%) - No: 34 (34.3%) - Uncertain: 2 (2%)   *Willing to pay for TUS*   - Yes: 60 (60.6%) - No: 28 (28.3%) - Uncertain: 11 (11%)   **Service quality**  Time to perform ultrasound  *Index test 1*   - 397.07 ± 192.34 seconds   *Index test 2*   - 355.63 ± 166.65 seconds   Quality of images  *Index test 1*  Comprehensive assessment score (n=99):   - Images were undiagnosable or not meaningful: 1 (1%) - Poor image quality may affect the diagnosis: 3 (3%) - Acceptable for interpretation: 20 (20.2%) - Minor suggestions for improvement of image quality: 50 (50.1 %) - Perfect: 25 (25.2%) - Total score:  3.96 ± 0.81   Qualification rate  Background image quality (n=99):   - Grayscale: 83 (83.8%) - Focus position: 89 (89.9%) - TGC: 94 (80.4%) - Depth: 58 (58.6%)   Target nodule image quality (n=56):   - Color Doppler adjustment: 52 (92.8%) - Visibility of all key information: 5 (94.6%)   *Index test 2*  Comprehensive assessment score (n=99):   - Images were undiagnosable or not meaningful: 1 (1%) - Poor image quality may affect the diagnosis: 20 (20.2%) - Acceptable for interpretation: 52 (52.5%) - Minor suggestions for improvement of image quality: 23 (23.2%) - Perfect: 3 (3%) - Total score:  3.07 ± 0.77   Qualification rate:  Background image quality (n=99):   - Gray value: 69 (69.6%) - Focus position: 85 (85.9%) - TGC: 68 (68.7%) - Depth: 24 (24.2%)   Target nodule image quality (n=38):   - Color Doppler adjustment: 29 (76.3%) - Visibility of all key information: 28 (73.7%)   Performers’ satisfaction rate  *Value of tele-US in diagnosis*   - Yes: 69/99 (69.7%) - No: 29/99 (29.3%) - Uncertain: 1/99 (1%)   *Guidance had a training effect on the performer*   - Yes: 68/99 (68%) - No: 29/99 (29.3%) - Uncertain: 2/99 (2%)   **Access to care**   - NR |
| **Ultrasound indication: Thyroid ultrasound** | | | | | | | |
| Author(s):   - Li et al. (2022)   Country(ies):   - China | Study design:   - Paired comparative accuracy study   Setting:   - Single center   Study period:   - April 2020 to June 2020 (3 months) | Recruitment:   - Total number of patients recruited: 99 - Excluded based on criteria: 2 - Declined to participate: 0 - Not approached: 0 - Attended: 97   *Index test 1*   - Number of patients:  97 - Dropouts: 0 - Analyzed: 97   *Index test 2*   - Number of patients: 97 - Dropouts: 0 - Analyzed: 97   *Reference test*   - Number of patients: 97 - Dropouts: 0 - Analyzed: 97 | **Tele-ultrasound**  Operator(s):   - Resident B   Real-time guidance:   - Yes   Mentors:   - Expert   Training:   - Lecture and Practice - Length: NR   Transmission:   - Real-time - Transmission bandwidth: high-speed network   Interpreter(s) of the results:   - Resident B and the remote expert through discussion | **In-person ultrasound by naive operator**  Operator(s):   - Resident A   Interpreter(s) of the results:   - Resident A | **In-person ultrasound by experts**  Number of References: 2   - The expert who guided resident B performed the on-site ultrasound and made independent interpretations - 2 other experts with 5 years of experience in thyroid ultrasound as off-site experts were designated to analyze all the data in a blind manner   Operator(s):   - The expert that guided resident B   Interpreter(s) of the results:   - The expert that guided resident B - 2 independent off-site US experts | - **Inter-operator consistency** between the two residents and the on-site US expert for thyroid was compared (Index test & Reference test + Index 2 & Reference test) - The background image quality - The target nodule image quality | **Patient care quality**  Proportion of agreement (on targeted nodules)  *Index test 1 & Reference test*: 59/66 (89.4%)  *Index test 2 & Reference test*: 39/66 (56.5%)  Sensitivity:  *Index test 1 & Reference test:* 59/66 (89.4%)  *Index test 2 & Reference test:* 39/66 (59.1%)  Specificity:  *Index test 1 & Reference test:* 24/31 (77.4%)  *Index test 2 & Reference test:* 1/31 (3.2%)  Positive Predictive Value (PPV):  *Index test 1 & Reference test*: 59/66 (89.4%)  *Index test 2 & Reference test:* 36/69 (56.5%)  Negative Predictive Value (NPV):  *Index test 1 & Reference test:* 24/31 (77.4%)  *Index test 2 & Reference test:* 1/28 (3.6%)  Intraclass Correlation Coefficient (ICC) (Interobserver agreement):  *Index test 1 & Reference:*  Target nodule features   - Composition: 0.819 (0.714-0.889) - Echogenicity: 0.694 (0.524-0.806) - Shape: 0.788 (0.668-0.868) - Margin: 0.657 (0.484-0.781) - Echogenic foci: 0.801 (0.686-0.877) - Vascularity: 0.775 (0.649-0.840)   ACR TI-RADS categories*:* 0.791 (0.672-0.870)  Target nodule measurement   - Transverse diameter: 0.979 (0.965-0.987) - Anterior-posterior diameter: 0.984 (0.9730.990) - Longitudinal diameter: 0.961 (0.935-0.976)   *Index test 2 & Reference*  Target nodule features   - Composition: 0.737 (0.552-0.853) - Echogenicity: 0.531 (0.263-0.723) - Shape: 0.392 (0.091-0.627) - Margin: 0.462 (0.175-0.676) - Echogenic foci: 0.602 (0.356-0.769) - Vascularity: 0.647 (0.420-0.798)   ACR TI-RADS categories*:* 0.724 (0.533-0.845)  Target nodule measurement*:*   - Transverse diameter: 0.972 (0.947-0.985) - Anterior-posterior diameter: 0.966 (0.937-0.982) - Longitudinal diameter: 0.964 (0.933-0.981)   Change in diagnosis/treatment /referral: NR  Patients’ satisfaction rate (n=97)  Synchronous TUS acceptance   - Yes: 60 (61.9%) - No: 32 (33.0%) - Uncertain: 5 (5.2%)   Willing to pay for TUS   - Yes: 58 (59.8%) - No: 35 (36.1%) - Uncertain: 4 (4.1%)   **Service quality**  Time to perform ultrasound:  *Index test 1*   - 274.40  ± 117.43 (range 110.00 to 706.00 seconds)   *Index test 2*   - 193.43  ± 63.93 (range 100.00 to 359.00 seconds)   Quality of images:  *Index test 1*  Comprehensive assessment score   - Poor and cannot be used for diagnosis: 1/97 (1%) - Not good enough and could affect the diagnosis: 4/97 (4%) - Flawed but can be used for diagnosis: 22/97 (22.7%) - Good and can be used for diagnosis with satisfaction: 45/97 (46.4%) - Excellent and can be used for diagnosis with a high level of satisfaction: 25/97 (23.7%) - Total score:  3.92 ± 0.86   Background image quality   - Gray value: 80 (82.5%) - Focus position: 87 (89.7%) - TGC: 78 (80.4%) - Depth: 83 (85.6%)   Target nodule image quality   - Color Doppler adjustment: 54 (91.5%) - Visibility of all key information: 56 (94.9%)   *Index test 2*  Comprehensive assessment score   - Poor and cannot be used for diagnosis: 2/97 (2.1%) - Not good enough and could affect the diagnosis: 23/97 (23.7%) - Flawed but can be used for diagnosis: 48/97 (49.5%) - Good and can be used for diagnosis with satisfaction: 19/97 (19.6%) - Excellent and can be used for diagnosis with a high level of satisfaction: 5/97 (5.2%) - Total score:  3.01 ± 0.85   Background image quality   - Gray value: 52 (53.6%) - Focus position: 78 (80.4%) - TGC: 37 (38.1%) - Depth: 65 (67%)   Target nodule image quality   - Color Doppler adjustment: 29 (74.4%) - Visibility of all key information: 27 (69.2%)   Performers’ satisfaction rate:  *Guidance is helpful*   - Yes: 61 (62.9%) - No: 34 (35.1%) - Uncertain: 2 (2.1%)   *Guidance had a training effect on the performer*   - Yes: 63 (64.9%) - No: 33 (34.0%) - Uncertain: 1 (1%)   **Access to care**   - NR |
| Author(s):   - Marini et al. (2021)   Country(ies):   - Peru + United States | Study design:   - Paired comparative accuracy study   Setting:   - Single center   Study period:   - June 2018 to March 2019 (10 months) | Recruitment:   - Total number of patients recruited: 121 - Excluded based on criteria: 0 - Declined to participate: 0 - Not approached: 0 - Attended: 121   *Index test 1*   - Number of patients: 121 - Dropouts: 0 - Analyzed: 121   *Index test 2*   - NA   *Reference test*   - Number of patients: 121 - Dropouts: 0 - Analyzed: 121 | **Tele-ultrasound (Volume sweep imaging – VSI)**  Operator(s):   - A nurse and a care technician   Real-time guidance:   - No   Mentors:   - NA   Training:   - Lecture and Practice - Length: 8 hours   Transmission:   - Storage - Transmission bandwidth: NR   Interpreter(s) of the results:   - Abdominal imaging attending radiologists | **NA** | **Standard of care ultrasound**  Operator(s):   - Peruvian Radiologists   Interpreter(s) of the results:   - Peruvian Radiologists | - **Agreement** between (VSI) and standard of care ultrasound on presence of thyroid nodules and lobe diameters (Index test 1 & Reference test) - Thyroid gland visualization and **image quality** of tele-ultrasound | **Patient care quality**  Proportion of agreement (Presence of a nodule): 98.3%  Sensitivity: NR  Specificity: NR  Positive Predictive Value (PPV): NR  Negative Predictive Value (NPV): NR  Kappa score (Presence of a nodule)   - k = 0.91 (0.78-1, p<0.0001)   Intraclass Correlation Coefficient (ICC) (thyroid lobe diameters)   - Right lobe AP: 0.37 (0.04-0.58, p=0.001) - Right lobe transverse: 0.57 (0.35-0.71, p <0.0001) - Left lobe AP: 0.42 (0.02-0.64, p<0.0001) - Left lobe transverse: 0.58 (0.01-0.79, p<0.0001) - Isthmus lobe AP: 0.48 (-0.22 to 0.77, p<0.0001)   Change in diagnosis/treatment /referral: NR  Patients’ satisfaction rate: NR  **Service quality:**  Time to perform ultrasound: NR  Image quality:  *Index test 1*  Image quality   - Acceptable 12.4% - Excellent 87.6%   Left lobe   - 100% studies had ≥80% visualization   Right lobe   - 88% studies had ≥80% visualization and 12% had 50-80% visualization   Isthmus   - 100% studies had ≥80% visualization   Performer’s satisfaction rate or confidence level: NR  **Access to care:**   - NR |
| **Ultrasound indication: Abdominal ultrasound** | | | | | | | |
| Author(s):   - Marini et al. (2021)   Country(ies):   - Peru + United States   . | Study design:   - Paired comparative accuracy study   Setting:   - Single center   Study period:   - June 2018 to March 2019 (10 months) | Recruitment:   - Total number of patients recruited: 144 - Excluded based on criteria: 0 - Declined to participate: 0 - Not approached: 0 - Attended: 144   *Index test 1*   - Number of patients: 144 - Dropouts: 0 - Analyzed: 144   *Index test 2*   - NA   *Reference test*   - Number of patients: 144 - Dropouts: 0 - Analyzed: 144   *Subpopulation*   - Examinations acceptable/excellent image quality - Number of patients: NR | **Tele-ultrasound (Volume sweep imaging – VSI)**  Operator(s):   - A nurse and a care technician   Real-time guidance:   - No   Mentors:   - NA   Training:   - Lecture and Practice - Length: 8 hours   Transmission:   - Storage - Transmission bandwidth: NR   Interpreter(s) of the results:   - Two separate board-certified abdominal fellowship-trained American radiologists | **NA** | **Standard of care ultrasound**  Operator(s):   - A Peruvian radiologist with 10 years of experience   Interpreter(s) of the results:   - Peruvian radiologist with 10 years of experience | - **Agreement** between VSI and standard of care ultrasound (Index test 1 & Reference test) - **Image quality** of VSI | **Patient care quality**  Proportion of agreement: ranging from 43.4-100%  *All exams*   - Liver Echogenicity: 99.3% - Liver Abnormal: 86.1% - Gallbladder: 70.1% - Pancreas Abnormal: 43.4% - Right Kidney Abnormal: 65.2% - Exam Abnormal: 94%   *Ignoring non-visualized cases*   - Liver Echogenicity: 99.3% - Liver Abnormal: 99.2% - Gallbladder: 92.7% - Pancreas Abnormal: 100% - Right Kidney Abnormal: 98.9% - Exam Abnormal: 94%   Sensitivity:   - Cholelithiasis: 84.2% (60.4 - 96.6%) - Cholelithiasis after consensus read: 89.5% (66.9 - 98.7%)   Specificity:   - Cholelithiasis: 97.7% (91.9 - 99.7%) - Cholelithiasis after consensus read: 97.7% (91.9 - 99.7%)   Positive Predictive Value (PPV): NR  Negative Predictive Value (NPV): NR  Kappa score:  *All exams*   - Liver Echogenicity: κ = 0.92 (0.84-1) - Liver Abnormal: κ = 0.15(-0.042-0.34) - Gallbladder: κ = 0.45(0.35-0.55) - Pancreas Abnormal: κ = 1 - Right Kidney Abnormal: κ = 0.13 (-0.11-0.37) - Exam Abnormal: κ = 0.84 (0.7-0.98)   *Ignoring non-visualized cases*   - Liver Echogenicity: 0.92(0.84-1) - Liver Abnormal: κ = 0.8(0.41-1.2) - Gallbladder: κ = 0.77(0.62-0.92) - Pancreas Abnormal: κ = 1 - Right Kidney Abnormal: κ = 0.66(0.033-1.3) - Exam Abnormal: κ = 0.79(0.65-0.93)   ***Subpopulation (Acceptable/excellent image quality exams)***  Proportion of agreement:  *All exams*   - Liver Echogenicity: 100% - Liver Abnormal: 98.9% - Gallbladder: 86.8% - Pancreas Abnormal: 100% - Right Kidney Abnormal: 86.2% - Exam Abnormal: 94.5%   *Ignoring non-visualized cases*   - Liver Echogenicity: 100% - Liver Abnormal: 98.9% - Gallbladder: 92.9% - Pancreas Abnormal: 100% - Right Kidney Abnormal: 98.7% - Exam Abnormal: 94.5%   Sensitivity:   - Cholelithiasis: 93.3% (68.1 - 99.8%) - Cholelithiasis after consensus read: 100% (78.2 - 100%)   Specificity:   - Cholelithiasis: 97.0% (89.5 - 99.6%) - Cholelithiasis after consensus read: 97.0 (89.5 - 99.6%)   Positive Predictive Value (PPV): NR  Negative Predictive Value (NPV): NR  Kappa score:  *All exams*   - Liver Echogenicity: κ = 1 (1-1) - Liver Abnormal: κ = 0.8 (0.41-1.2) - Gallbladder: κ = 0.69 (0.55-0.83) - Pancreas Abnormal: κ = 1 - Right Kidney Abnormal: κ = 0.13 (-0.11-0.37) - Exam Abnormal: κ = 0.84 (0.7-0.98)   *Ignoring non-visualized cases*   - Liver Echogenicity: κ = 1 (1-1) - Liver Abnormal: κ = 0.8 (0.41-1.2) - Gallbladder: κ = 0.8 (0.65-0.95) - Pancreas Abnormal: κ = 1 - Right Kidney Abnormal: κ = 0.66 (0.033-1.3) - Exam Abnormal: κ = 0.84 (0.7-0.98)   Intraclass Correlation Coefficient (ICC): NR  Change in diagnosis/treatment /referral: NR  Patients’ satisfaction rate: NR  **Service quality:**  Time to perform ultrasound:   - Approximately 10 minutes   Image quality:   - Excellent: 24.3% (17.6-32.1%) - Acceptable: 38.9% (30.9-47.4%) - Poor: 36.8% (28.9%-45.2%)   Performer’s satisfaction rate or confidence level: NR  **Access to care**   - NR |
